# Microvillar and ciliary defects in zebrafish lacking an actin-binding bioactive peptide amidating enzyme

**DOI:** 10.1038/s41598-018-22732-9

**Published:** 2018-03-14

**Authors:** Dhivya Kumar, Rebecca T. Thomason, Maya Yankova, Jonathan D. Gitlin, Richard E. Mains, Betty A. Eipper, Stephen M. King

**Affiliations:** 1 Department of Molecular Biology and Biophysics, University of Connecticut Health Center, Farmington, CT 06030 USA; 2 Eugene Bell Center for Regenerative Biology and Tissue Engineering, Marine Biological Laboratory, Woods Hole, MA 02543 USA; 3 Department of Neuroscience, University of Connecticut Health Center, Farmington, CT 06030 USA; 4 Electron Microscopy Facility, University of Connecticut Health Center, Farmington, CT 06030 USA; 5 Present Address: Department of Biochemistry and Biophysics, University of California San Francisco, San Francisco, CA 94158 USA; 6 Present Address: University of Virginia, Charlottesville, VA 22904 USA

## Abstract

The assembly of membranous extensions such as microvilli and cilia in polarized cells is a tightly regulated, yet poorly understood, process. Peptidylglycine α-amidating monooxygenase (PAM), a membrane enzyme essential for the synthesis of amidated bioactive peptides, was recently identified in motile and non-motile (primary) cilia and has an essential role in ciliogenesis in *Chlamydomonas*, *Schmidtea* and mouse. In mammalian cells, changes in PAM levels alter secretion and organization of the actin cytoskeleton. Here we show that lack of Pam in zebrafish recapitulates the lethal edematous phenotype observed in *Pam*^*−/−*^ mice and reveals additional defects. The *pam*^*−/−*^ zebrafish embryos display an initial striking loss of microvilli and subsequently impaired ciliogenesis in the pronephros. In multiciliated mouse tracheal epithelial cells, vesicular PAM staining colocalizes with apical actin, below the microvilli. In PAM-deficient *Chlamydomonas*, the actin cytoskeleton is dramatically reorganized, and expression of an actin paralogue is upregulated. Biochemical assays reveal that the cytosolic PAM C-terminal domain interacts directly with filamentous actin but does not alter the rate of actin polymerization or disassembly. Our results point to a critical role for PAM in organizing the actin cytoskeleton during development, which could in turn impact both microvillus formation and ciliogenesis.

## Introduction

Membranous cellular extensions such as cilia and microvilli play key roles in development, cellular homeostasis and/or the generation of propulsive force and fluid flow. The assembly of these structures in ciliated epithelia lining the trachea, ventricles and kidneys in various organisms requires cellular polarization and the coordinated actions of the actin cytoskeleton, microtubular network and vesicular trafficking machinery^1^.

Microvilli are actin-rich protrusions that greatly increase the absorptive, sensory and secretory surface area of epithelial cells^2,3^. Actin filaments in the microvillar core are bundled together by crosslinking proteins (*e.g*. villin, fimbrin and epsin), while other proteins (*e.g*. ezrin and myosin-1a) link actin bundles to the plasma membrane^3,4^. Cilia are microtubule-based sensory and motile organelles that extend from most cells in humans. Multiple motile cilia in the respiratory epithelium and brain ependymal cells propel fluids (such as mucus and cerebrospinal fluid); in other cell types, immotile (primary) cilia act as sensors of the extracellular environment and as dedicated signaling compartments^5^. Defects in ciliary assembly/function lead to a group of heterogeneous, multisystemic disorders collectively termed ciliopathies^6^; phenotypes include severe brain, skeletal and heart abnormalities, infertility, anosmia and laterality defects. The structural components of microvilli are well characterized, but the mechanisms by which cells regulate their assembly and maintenance are unclear. Likewise, although a considerable amount is known about ciliary assembly, how this process is initiated and how the trafficking of membrane components is controlled remain poorly understood.

Peptidylglycine α-amidating monooxygenase (PAM) is a bifunctional enzyme required for one of the last steps in the biosynthesis of bioactive peptides such as oxytocin, vasopressin, gastrin and neuropeptide Y, and is thus key to peptidergic signaling^7^. PAM-null mice display severe edema and vasculature defects, and do not survive beyond embryonic day E14.5^8^. Pam-null *Drosophila* die while molting to the second larval instar^9^. The enzymatic domains of PAM (peptidylglycine α-hydroxylating monooxygenase [PHM] and peptidyl-α-hydroxyglycine α-amidating lyase [PAL]) reside in the secretory pathway lumen, where they act sequentially to convert inactive peptide precursors with a C-terminal glycine residue into amidated bioactive peptide products that are subsequently secreted^7^. The cytosolic C-terminal domain (CD) of PAM, a type I integral membrane protein, is essential for trafficking through the secretory and endocytic pathways^10^. This unstructured domain interacts with Kalirin and Trio^11^, GDP/GTP exchange factors (GEFs) for Rac1, RhoG and RhoA, small GTPases that regulate actin filament dynamics, and with KIS/Uhmk1, a Ser/Thr kinase that interacts with stathmin, a regulator of microtubule depolymerization^11^. These cytosolic interactors are thought to contribute to the ability of exogenous PAM to alter cytoskeletal organization and inhibit regulated peptide secretion in a neuroendocrine cell line^12^.

Apart from its well-recognized role in the neuroendocrine system, PAM is also present in cilia^13^, and has an evolutionarily conserved role in ciliogenesis^14^. PAM-deficient *Chlamydomonas* cells are unable to assemble cilia beyond the transition zone and have altered levels and localizations of several ciliary proteins. RNAi-mediated knockdown of PAM expression in the planarian *Schmidtea mediterranea* leads to severe loss of motile cilia on the ventral surface due to defective ciliary remodeling, and primary cilia develop abnormally in the neuroepithelium of *Pam*^*−/−*^ mouse embryos^14^. However, the mechanism(s) by which PAM regulates ciliary assembly in these diverse cell types is unknown. To further investigate the roles of PAM, we turned to zebrafish, a genetically tractable model system for analyzing the role of ciliogenesis in early vertebrate development^15^. For example, motile ciliary dysfunction in zebrafish can result in hydrocephalus, laterality defects, curved body axis, kidney cysts, and ectopic otoliths in the otic vesicle^16–24^. As in other vertebrates, multiple amidated peptides have been identified in the zebrafish brain^25^.

The actin cytoskeleton is intimately involved in ciliogenesis and indeed branched actin polymerization has been shown to antagonize ciliary elongation^26–28^. Actin and its regulators control ectocytosis from cilia^29^, and actin polymerization can lead to “decapitation” of the ciliary tip^30^. Furthermore, cytochalasin D treatment of multiciliated cells disrupts the apical actin web, leading to defects in basal body docking and ciliary motility^31^. In this study, we demonstrate that lack of Pam in zebrafish leads to the loss of both cilia and microvilli in the pronephros, suggesting an essential connection between this amidating enzyme and the actin cytoskeleton. We also find that PAM colocalizes with actin but not microvilli in polarized multi-ciliated murine tracheal epithelial cells, and use *in vitro* biochemistry to demonstrate a direct interaction between rat PAM-CD and filamentous actin. Furthermore, analysis of PAM-deficient *Chlamydomonas* reveals that the PAM-actin connection has deep evolutionary roots. Together, data from these three model systems support a key conserved role for PAM-actin associations in building cytoskeleton-based membranous extensions in diverse cell types.

## Results

### Pam is expressed in ciliated tissues of zebrafish embryos

**Figure 1:**
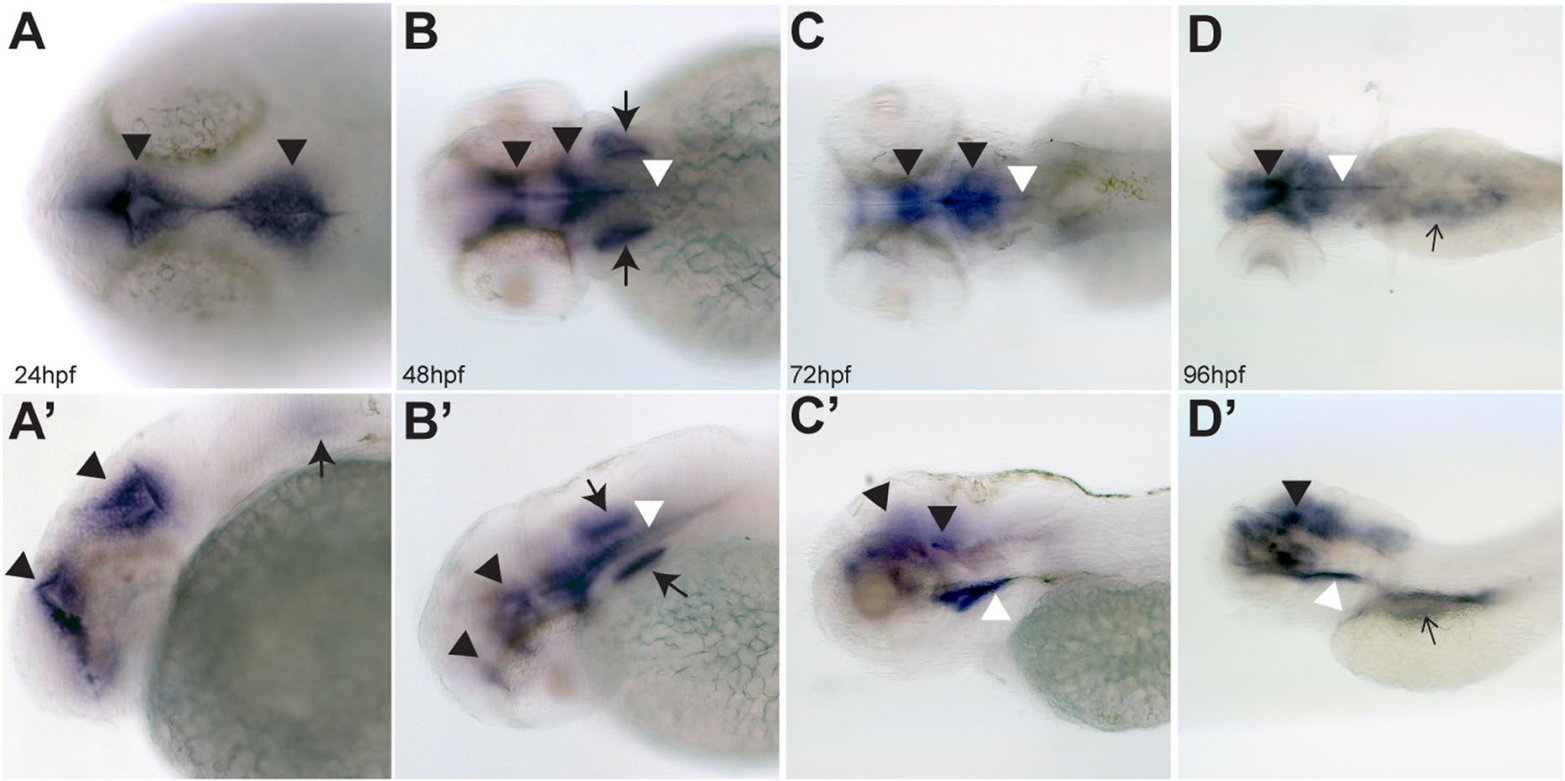
PAM expression in zebrafish embryos. Dorsal (**A**–**D**) and lateral (**A’–D’**) views of zebrafish embryos at 24, 48, 72 and 96 hpf visualized by *in situ* hybridization with an antisense probe for *pam*; staining with the sense control probe using the same processing conditions is shown in Supplemental Fig. S1. *pam* mRNA expression is detected in the ependyma lining the developing ventricles (black arrowheads), otic vesicles (thick arrows), floor plate (white arrowheads), and was also diffusely abdominal (thin black arrows in panels **D** and **D’**). No *pam* expression was evident in the eye or cardiac tissue.

Wildtype zebrafish embryos were examined by *in situ* hybridization using a *pam* antisense probe (Fig. 1); the control (sense) probe showed no signal (Supplemental Fig. S1). At early developmental time points (24 hours post-fertilization (hpf)), *pam* transcripts were detected in cells lining the brain ventricles (Fig. 1A,A’) and subsequently, at 48 hpf, in the otic vesicles (Fig. 1B,B’) and an elongated structure consistent with the floor plate (Fig. 1B,B’), where it is also expressed in rats^32^. At a later timepoint (96 hpf), expression in the general area of the developing pronephros and gut became evident (Fig. 1D,D’). Thus, there is a strong correlation between the expression of *pam* and the development of ciliated tissues in zebrafish. Interestingly, although *Pam* mRNA is highly expressed in heart tissue in mice^32,33^, no cardiac staining was observed in zebrafish.

### Generation of Pam-null zebrafish

**Figure 2:**
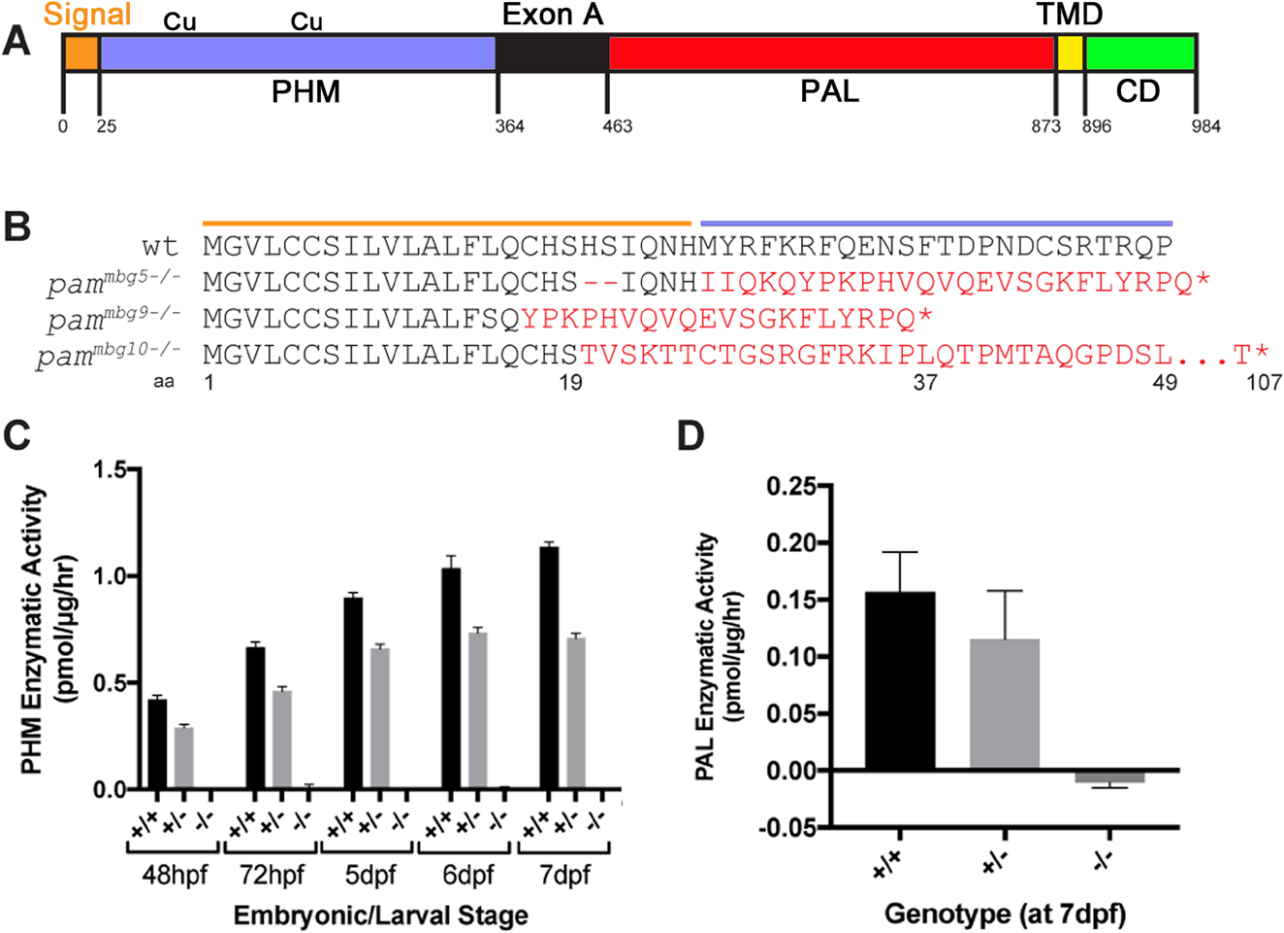
Generation of *pam*^−/−^ zebrafish. (**A**) Schematic depicting the domains in zebrafish Pam protein. The signal sequence (orange), PHM domain (purple) with two copper-binding sites, linker region (Exon A; black), PAL catalytic domain (red), transmembrane domain (TMD; yellow) and cytosolic domain (CD; green) are shown; residue numbers indicate the boundaries corresponding to each domain. (**B**) Predicted protein sequences for three *pam* mutant lines generated through CRISPR-Cas9 genome editing; frame-shift mutations (in red) result in truncation of all three proteins before the beginning of the PHM domain. The colored lines indicate the signal sequence (orange) and beginning of the PHM domain (blue). (**C**) PHM enzyme assays of embryos collected at the indicated developmental stages from wildtype siblings (*pam*^+/+^) and both heterozygous (*pam*^*mbg5*+/−^) and homozygous (*pam*^*mbg5*−/−^) *pam*^*mbg5*^ animals. At all stages, the homozygous mutant had no detectable PHM activity. Data plotted as mean ± SD (n = 3). (**D**) PAL enzyme activity in *pam*^+/+^, *pam*^*mbg5*+/−^ and *pam*^*mbg5*−/−^ 7 dpf zebrafish embryos; no PAL activity was detected in the homozygous *pam*^*mbg5*−/−^ embryos. Data plotted as mean ± SD (n = 3).

The zebrafish genome contains a single *pam* gene (ZDB-GENE-090313-384) that yields two mRNA splice variants encoding Pam proteins that differ only in the PAL domain (UniProt A0A0R4IFY7 and A0A0R4IIV2), and a third non-protein coding processed transcript (ENSDART00000157885.1). Both Pam isoforms consist of an N-terminal signal sequence, followed by the canonical bifunctional enzymatic cores (PHM and PAL), a single-pass transmembrane domain and a cytosolic C-terminal domain (Fig. 2A). This same domain organization for membrane-PAM has been conserved throughout the metazoa (except insects, which express only separate PHM and PAL proteins), and is even found in chlorophyte green algae such as *Chlamydomonas*^13,34^.

To more fully examine the role of PAM during vertebrate development and in the assembly of cilia and actin-based structures, we generated three CRISPR/Cas9-mediated *pam*-mutant zebrafish lines^35,36^. These alleles (designated *pam*^*mbg5*^, *pam*^*mbg9*^ and *pam*^*mbg10*^) introduce deletions/insertions into exon 1, leading to altered protein sequences after residues 18, 16 and 19, which are all located within the signal sequence (Fig. 2B). Additional unrelated sequences of 30, 20 and 87 residues, respectively, occur before a stop codon is reached (Fig. 2B and Supplemental Fig. S2A); the *pam*^*mbg5*^ line was used for most of the subsequent phenotypic analyses. Sequence and PCR confirmation of the heterozygous (*pam*^*mbg5*+/−^) and homozygous (*pam*^*mbg5*−/−^) embryos is shown in Supplemental Figs S2B,C. The genotypes of five randomly selected clutches of embryos derived from *pam*^*mbg5*+/−^ × *pam*^*mbg5*+/−^ crosses were examined at different developmental stages; embryos were obtained at approximately the expected normal Mendelian ratios (Table 1).

**Table 1:** Genotypes obtained from *pam*^*mbg5*+/−^ × *pam*^*mbg5*+/−^ crosses.

**Clutch Number**	**Stage Analyzed**	**Genotype**	**n (%)**	χ^2^ **Test for Expected Mendelian Ratios**
1	48 hpf	+/+ +/− −/−	24 (23%) 54 (51%) 28 (26%)	0.34 (P < 0.9 but >0.5)
2	72 hpf	+/+ +/− −/−	26 (27%) 54 (57%) 16 (17%)	3.57 (P < 0.5 but >0.1)
3	5 dpf	+/+ +/− −/−	21 (22%) 54 (57%) 19 (20%)	2.17 (P < 0.5 but >0.1)
4	7 dpf	+/+ +/− −/−	17 (24%) 40 (57%) 13 (19%)	3.86 (P < 0.5 but >0.1)
5	7 dpf	+/+ +/− −/−	8 (27%) 17 (57%) 5 (17%)	1.13 (P < 0.9 but >0.5)

The genotypes of five clutches of embryos from *pam*^*mbg5*+/−^ × *pam*^*mbg5*+/−^ crosses were determined. Wildtype, heterozygous and homozygous mutant siblings were obtained at approximately the expected Mendelian ratios for all stages examined from 48 hpf to 7 dpf (days post-fertilization); for the five clutches combined, χ^2^ = 5.58, DF = 2, P < 0.1 but >0.05. Percentages do not always sum to 100% due to rounding.

Hydroxylation of the Cα atom of the C-terminal glycine of a peptide precursor by PHM is absolutely required for the PAM-mediated amidation reaction to proceed, while cleavage of the N-C bond attacked by PAL can occur spontaneously under certain conditions^7,37^. To demonstrate that these are true null alleles, we measured the PHM enzymatic specific activity in lysates prepared from wildtype siblings, *pam*^*mbg5*+/−^ heterozygotes and *pam*^*mbg5*−/−^ homozygotes at five embryonic/larval stages. PHM specific activity increased steadily in wildtype zebrafish embryos until 7 dpf. The homozygous mutant strain exhibited no detectable PHM activity at any developmental stage tested (Fig. 2C); thus, there are no maternally-derived stores of this enzyme at 48 hpf or beyond. We consistently observed that the PHM specific activity in lysates of heterozygous animals was ~75% that of wildtype siblings (Fig. 2C). Similarly, *pam*^*mbg5*−/−^ homozygotes had no detectable PAL activity at 7 dpf, whereas the *pam*^*mbg5*+/−^ heterozygotes again exhibited ~75% of wildtype specific activity (Fig. 2D). The increased PHM and PAL specific activity observed in heterozygotes suggests that Pam expression from the remaining wildtype allele may be upregulated. In contrast, similarly enhanced enzymatic activity was not observed in adult *Pam*^+/−^ heterozygous mice^8^.

### *pam*^−/−^ zebrafish embryos display multiple cilia-related phenotypes

**Figure 3:**
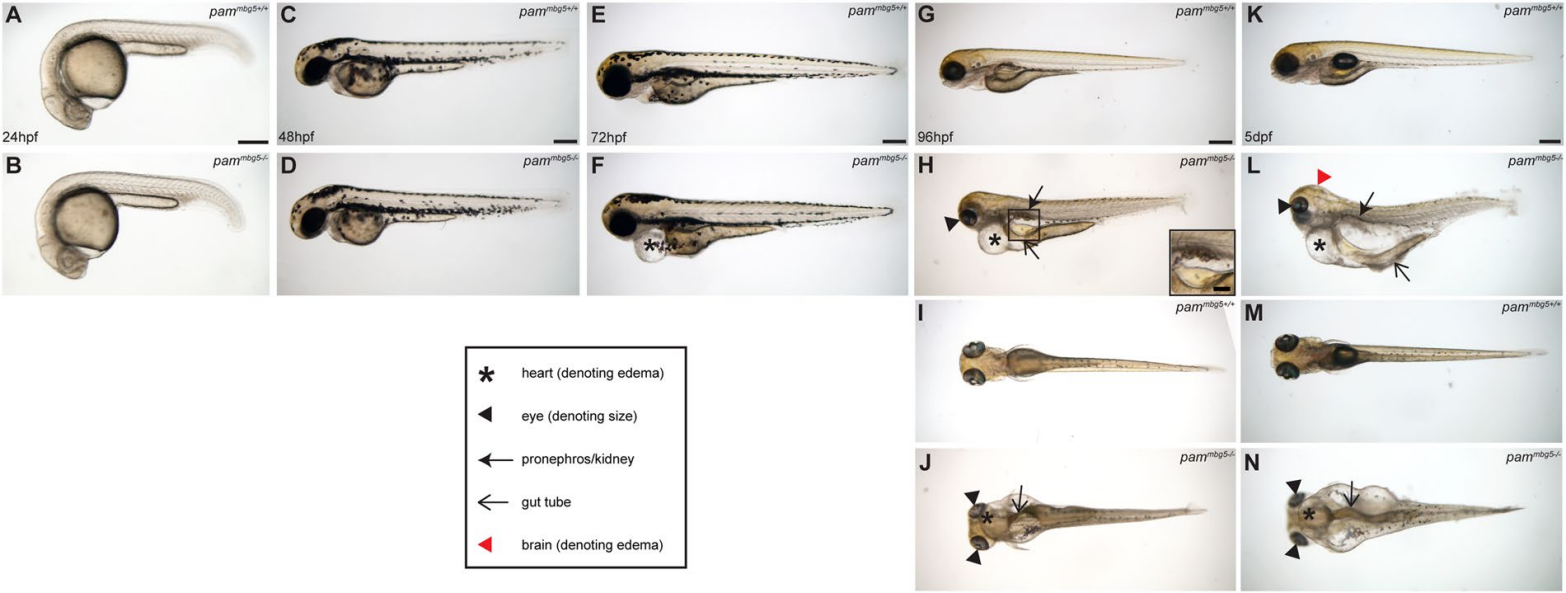
Phenotypic analysis of *pam*^−/−^ zebrafish embryos. Dorsal and lateral views of wildtype (panels **A**,**C**,**E**,**G**,**I**,**K** and **M**) and *pam*^*mbg5−/−*^ (panels **B**,**D**,**F**,**H**,**J**,**L** and **N**) embryos; comparison of wildtype (**A** and **C**) and *pam*^*mbg5−/−*^ mutants (**B** and **D**) revealed no obvious differences at 24 and 48 hpf. By 72 hpf, *pam*^*mbg5*−/−^ embryos develop pericardial edema (asterisk in **F**); by 96 hpf, additional edema around the abdomen (thin arrows in **H** and **J**), irregular cyst-like protrusions in the abdominal region (inset in **H**), and smaller eyes (arrowheads in **H** and **J**) were observed. The general area of the pronephros is indicated by thick arrows (in **H** and **L**). Severe edema developed at 5 dpf in *pam*^*mbg5*−/−^ mutants (**L** and **N**). Additional edema in the brain was also visible (red arrowhead in **L**). Developmentally normal wildtype sibling controls at 5 dpf are shown for comparison (**K** and **M**). A key to the symbols used is provided.

Pam-null zebrafish embryos are phenotypically indistinguishable from their wildtype siblings until 48 hpf (Fig. 3A–D), and have normal *situs*; unlike many ciliary mutants and morphants which exhibit laterality defects, no laterality defects were detected in 300 embryos from 3 clutches showing normal Mendelian ratios. As Pam is highly expressed in the otic vesicles, we examined these organs in the *pam*^−/−^ embryos and found that otoliths formed, and that both kinocilia and stereocilia were present (Supplemental Fig. S3E–H). Similarly, cilia were present on the olfactory bulb and neuromast cells of *pam*^−/−^ embryos (Supplemental Fig. S3A–D). Although Pam is not expressed in the heart at this stage, cardiac edema was apparent at 72 hpf in *pam*^−/−^ embryos (Fig. 3F compared to 3E). At 96 hpf, the *pam*^−/−^ homozygotes developed irregular cyst-like protrusions in the abdominal region (inset in Fig. 3H and Supplemental Fig. S4) and their eye size was noticeably smaller (Fig. 3H,J) compared to wildtype siblings (Fig. 3G). Dorsal views of control (Fig. 3I) and *pam*^−/−^ (Fig. 3J) embryos showed increased edema around the heart and in the abdomen of the homozygous mutant animals (Fig. 3J). At 5 dpf, *pam*^−/−^ zebrafish developed severe edema around the heart and in the abdomen (Fig. 3L,N, respectively) compared to controls (Fig. 3K,M). Some mild hydrocephalus was also visible at this stage (Fig. 3L), and the difference in eye size was more obvious (Fig. 3L,N). By approximately 10 dpf, all the *pam*^−/−^ zebrafish embryos were unable to swim and ultimately died, presumably due to the massive edema that resulted from pronephric and cardiac dysfunction. This edematous lethal phenotype showed 100% penetrance.

**Figure 4:**
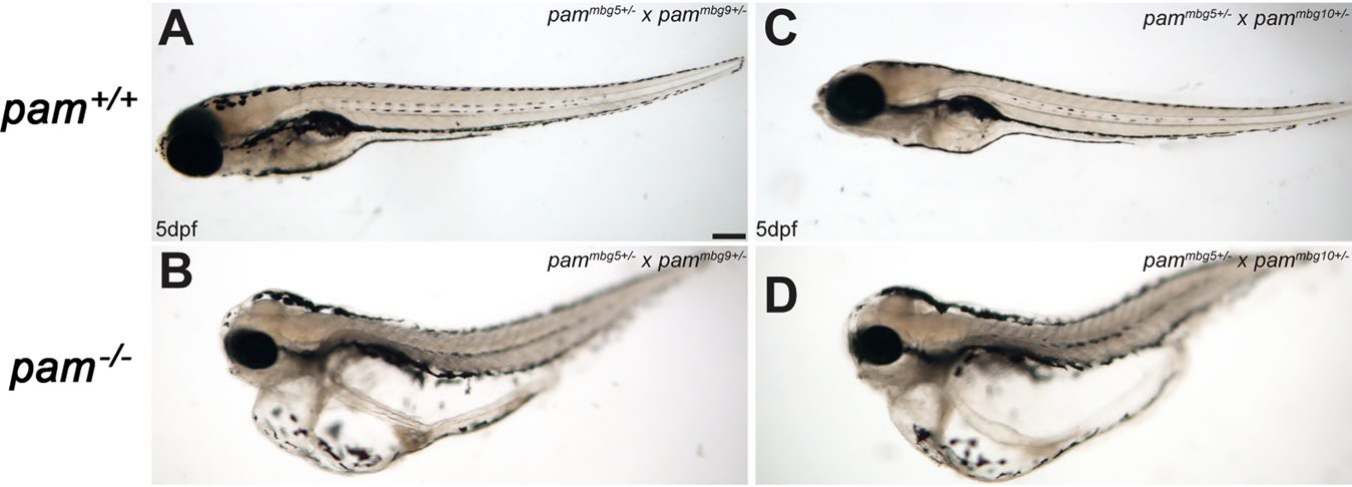
Heteroallelic *pam*^−/−^ zebrafish display severe edema. Heterozygous zebrafish bearing different *pam*^*mbg*^ alleles were crossed and the phenotypes of the resulting *pam*^−/−^ heteroallelic embryos and their wildtype siblings were examined at 5 dpf. Wildtype *pam*^+/+^ embryos developed normally (**A**,**C**). In contrast, heteroallelic *pam*^−/−^ homozygotes (*pam*^*mbg5*^/*pam*^*mbg9*^ and *pam*^*mbg5*^*/pam*^*mbg10*^) derived from *pam*^*mbg5*+/−^ × *pam*^*mbg9*+/−^ and *pam*^*mbg5*+/−^ × *pam*^*mbg10*+/−^ crosses exhibited severe edema (**B**,**D**).

To examine whether these phenotypes derive solely from the loss of Pam, we performed a genetic complementation test. Heterozygotes bearing different *pam*^*mbg*^ alleles were crossed (*pam*^*mbg5*+*/*−^ × *pam*^*mbg9*+*/*−^ and *pam*^*mbg5*+/−^ × *pam*^*mbg10*+/−^) and the *pam*^+/+^ wildtype and *pam*^−/−^ heteroallelic mutant siblings were examined at 5 dpf (Fig. 4). Homozygous *pam*^−/−^ mutants bearing either *pam*^*mbg5*^/*pam*^*mbg9*^ (Fig. 4B) or *pam*^*mbg5*^*/pam*^*mbg10*^ (Fig. 4D) alleles both showed the severe edematous phenotype observed in *pam*^*mbg5*−/−^ embryos, whereas the wildtype siblings were developmentally normal (Fig. 4A,C, respectively). Therefore, complementation did not occur, and the defects observed derive from the loss of Pam function rather than being caused by an off-target effect in the *pam*^*mbg5*^ CRISPR/Cas9-derived mutant line.

Thus, zebrafish lacking Pam recapitulate the edematous phenotype observed in *Pam*^−/−^ mouse embryos at E14.5^8^. Use of the zebrafish system revealed additional defects in the kidney, eyes and brain. Development of fluid-filled cysts in the kidney, hydrocephalus and edema would be consistent with a defect in ciliary function^16^.

### *pam*^−/−^ zebrafish embryos lack microvilli in the pronephros and exhibit defects in ciliogenesis

**Figure 5:**
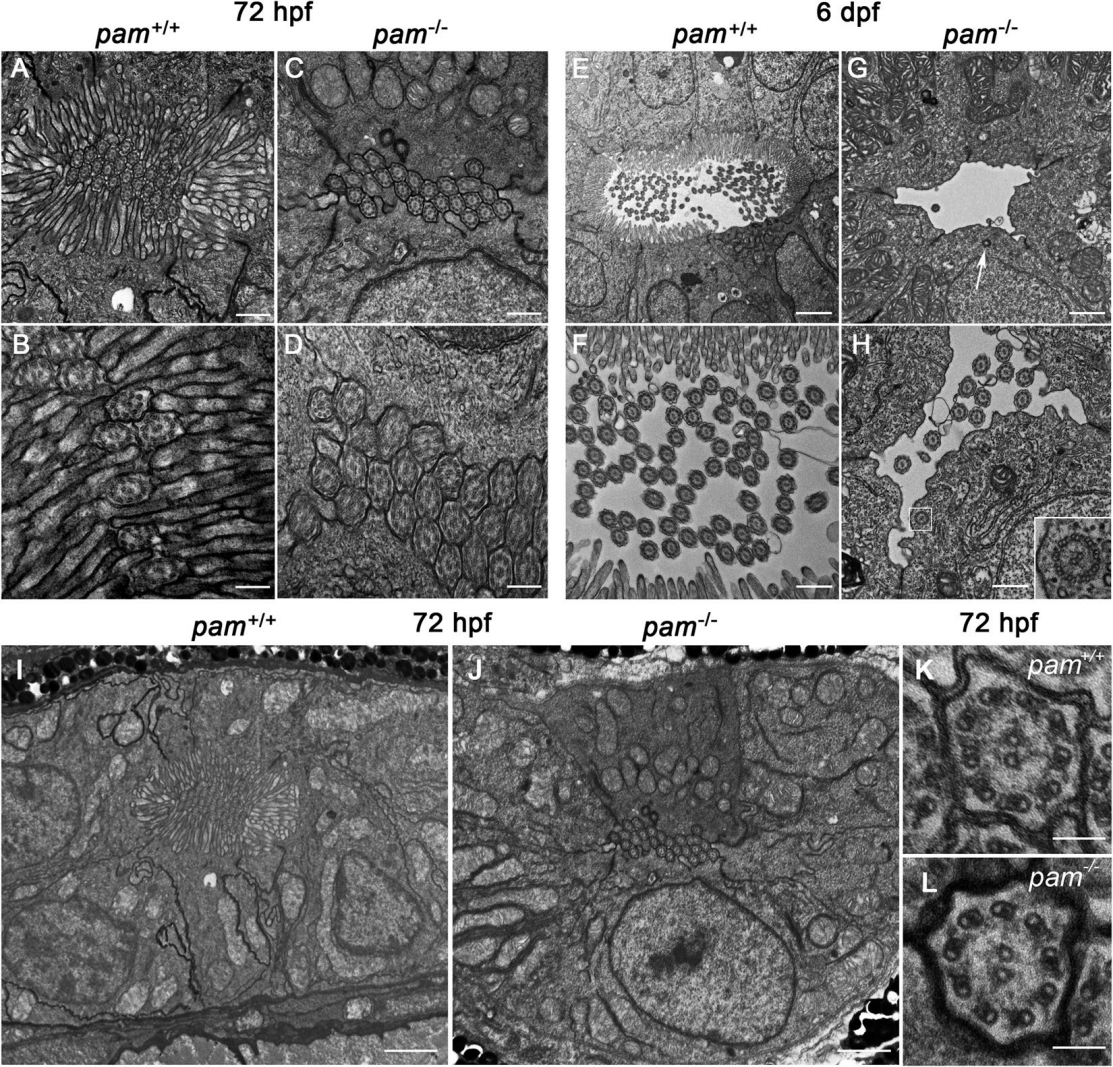
*pam*^−/−^ zebrafish exhibit microvillar and ciliary assembly defects in the pronephros. Transmission electron micrographs of transverse sections through the pronephros of *pam*^−/−^ embryos and their wildtype siblings at 72 hpf (**A**–**D** and **I**,**J**) and 6 dpf (**E**–**H**); the images shown were taken from approximately midway along the pronephros. At 72 hpf, the pronephros lumen of wildtype zebrafish is occluded with numerous cilia surrounded by a dense array of microvilli (**A**,**B**). Although densely packed cilia were evident in the pronephric lumen of *pam*^−/−^ embryos at this stage, brush border microvilli were absent (**C**,**D**). At 6 dpf, the lumen of the wildtype zebrafish pronephros was more open; numerous cilia appeared in the lumen, which was surrounded by epithelial cells extending a dense array of microvilli (**E**,**F**). In contrast, the lumen of the *pam*^−/−^ pronephros showed a severe reduction in the number of cilia along with the continued absence of a brush border (**G**,**H**). Occasional cytosolic axonemes (inset in **H**) and undocked centrioles/basal bodies (arrow in G and see Supplemental Fig. S6) were observed in the epithelial cells lining the pronephric duct of the *pam*^−/−^ embryos. Lower magnification images (**I**,**J**) of the region surrounding the pronephros of the 72 hpf embryos shown in panels (**A**,**B**) illustrate the general cellular architecture. The cilia present in *pam*^−/−^ embryos at 72 hpf were morphologically normal (**K**,**L**). Bars = 100 nm (**K**,**L**), 500 nm (**A**,**C**,**F**,**H**), 250 nm (**B**,**D**), 1 μm (**G**) and 2 μm (**E**,**I**,**J**).

To further assess whether cilia were defective in the *pam*^−/−^ embryos, we used transmission electron microscopy to examine the architecture of the pronephros at 72 hpf, before kidney cysts are apparent in mutant embryos, and at 6 dpf, well after kidney cysts appeared (Fig. 5A–H). In control embryos at 72 hpf (Fig. 5A,B,I), the pronephros lumen was occluded by a dense array of apical microvilli surrounding tightly packed cilia located in the center of the lumen. The pronephros in the *pam*^−/−^ zebrafish embryos also contained numerous tightly packed cilia; however, we observed a striking loss of brush border microvilli (Fig. 5C,D,J) compared to controls (Fig. 5A,B,I). The cilia in *pam*^−/−^ mutants at this stage had normal ultrastructure (Fig. 5C,D,K,L).

At 6 dpf, the pronephric lumen in control embryos was more open, containing numerous cilia surrounded by apical brush border microvilli extending from the epithelial cells (Fig. 5E,F and Supplemental Fig. S5). In the more posterior region, there were fewer microvilli and cilia (Supplemental Fig. S5E). In contrast, although microvilli and cilia were present in the most anterior region of the pronephros of *pam*^−/−^ animals (Supplemental Fig. S5F), much of the lumen was empty; the apical surface of the epithelial cells lacked microvilli and few cilia were present. The lumen was varyingly shaped, with the more posterior regions significantly more open compared to wildtype siblings (Fig. 5G,H and Supplemental Fig. S5G–J,M and N). Together these data revealed that the pronephros lacked brush border microvilli throughout most of its length in *pam*^−/−^ zebrafish, and that the loss of microvilli preceded ciliary loss.

At 6 dpf we occasionally observed axonemes that had assembled in the cytoplasm of cells lining the mutant embryo pronephros (Fig. 5H and inset). These ectopically localized axonemes were of normal morphology but lacked a ciliary membrane (inset in Fig. 5H); strikingly, this unusual cytosolic axoneme assembly phenotype was observed previously in PAM-deficient planaria^14^. The occurrence of ectopic cytosolic axonemes lacking a ciliary membrane points to a basal body docking and/or membrane trafficking defect in these mutant embryos. Indeed, occasional undocked centrioles/basal bodies were evident in the cytoplasm of the *pam*^−/−^ pronephric epithelial cells (arrow in Fig. 5G and Supplemental Fig. S6).

### PAM co-localizes with apical actin in ciliated airway epithelial cells

**Figure 6:**
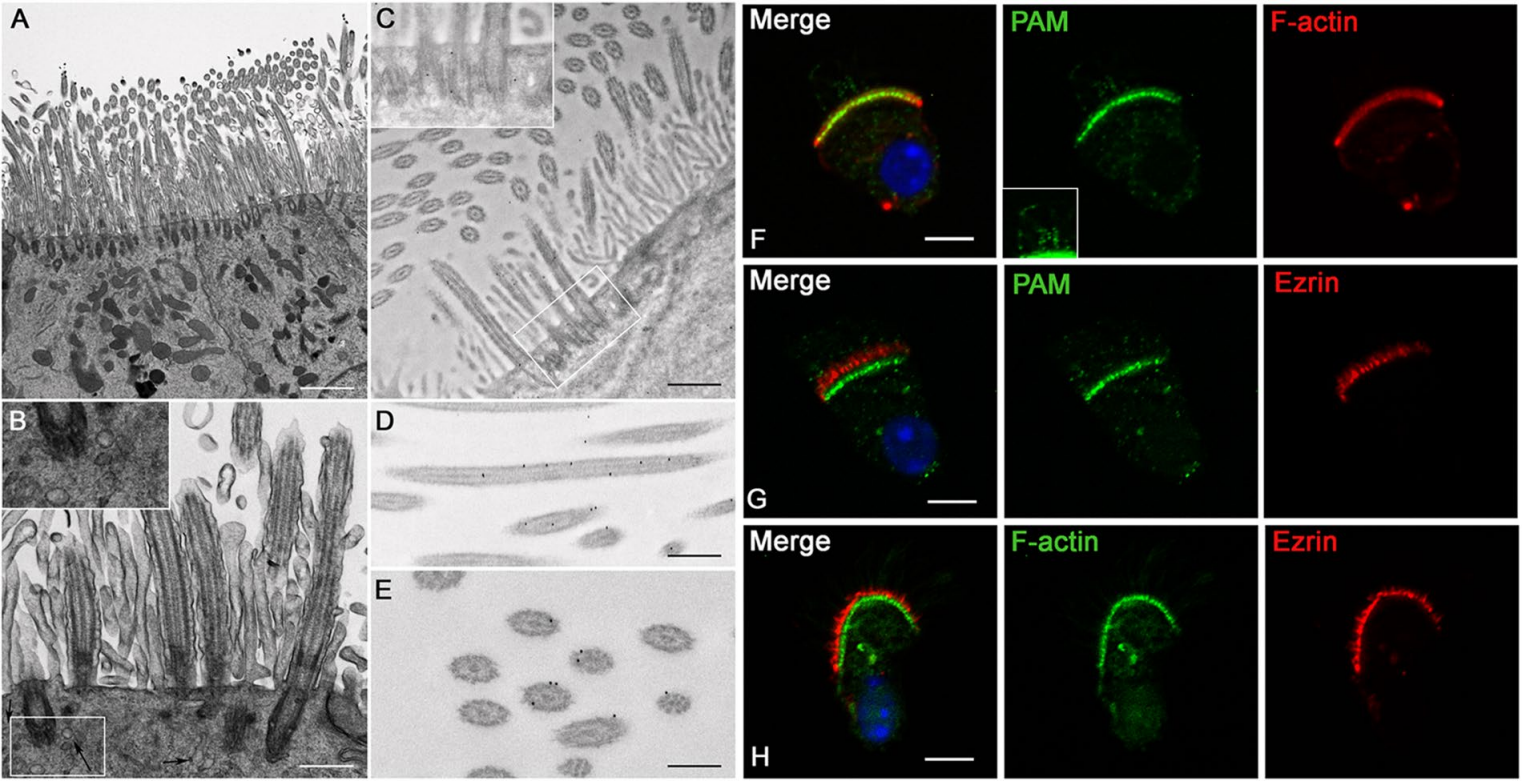
PAM colocalizes with apical actin and cilia in tracheal epithelial cells. Transmission electron micrographs of wildtype murine tracheal epithelium revealed multiciliated cells with a dense array of microvilli extending from the apical surface (**A**,**B**). Numerous membranous structures were readily detected near the basal bodies, which were docked at the plasma membrane (arrows and inset in **B**). Immunogold electron microscopy indicated that PAM was present in the peri-basal body region of tracheal cells (**C**), and was closely associated with cilia (**D**,**E**). Immunostaining of airway epithelial cells with Bodipy-phalloidin (red) and PAM antibody (green) (**F**); PAM (green) and ezrin (red) (**G**); and FITC-phalloidin (green) and ezrin (red) (**H**). The inset in (**F**) shows the ciliary PAM signal. Note that PAM staining in these cells was previously shown to be abolished by treatment with the antigenic peptide^13^. Bars = 2 μm (**A**), 500 nm (**B**–**D**), 250 nm (**E**), and 5 μm (**F**–**H**).

As the available PAM antibodies were raised against rat PAM and do not detect the zebrafish protein (which shares 54% identity), we turned to ciliated mouse tracheal epithelial cells, which are also highly polarized with cilia and microvilli at their apical surface, to examine the localization of PAM (Fig. 6A,B). Transmission electron microscopy revealed a complex array of interdigitating cilia and microvilli. The apical cytoplasm surrounding the basal bodies was enriched in membranous structures indicative of vesicular trafficking (Fig. 6B, inset).

We previously showed that PAM localizes in foci along the length of cilia in airway epithelial cells, and near the base of the cilia, adjacent to the basal bodies^13^. Therefore, we next used immunoelectron microscopy to further explore the location of PAM near the apical surface of these cells (Fig. 6C–E). PAM staining was most intense in the basal body region (Fig. 6C), presumably associated with the membranous structures identified there (inset in Fig. 6B). Consistent with our earlier study^13^, gold particles were also found along the ciliary length (Fig. 6C,D). In cross-sections of cilia, it was apparent that gold particles were present on or near the external face of the microtubular axoneme (Fig. 6E). In contrast, almost no gold particles were associated with microvilli.

Since basal bodies are closely associated with the actin cytoskeleton in polarized tracheal epithelial cells^38^, we co-labeled airway epithelial cells with fluorescent phalloidin, which preferentially binds filamentous actin, and antibodies to PAM (Fig. 6F). PAM colocalized with the fluorescently-tagged phalloidin, near the apical surface of these cells; punctate PAM staining was also observed in cilia (and see ref.^13^). To determine whether this staining corresponded to the apical actin web or the cell-proximal region of the microvilli themselves, we utilized an antibody to ezrin, a component of the microvillar core, and an antibody to PAM. Ezrin staining was apical to PAM staining (Fig. 6G); ezrin was predominantly localized in microvilli which were not detected strongly by phalloidin (Fig. 6H). Collectively, these results suggest a close association of PAM with the apical actin network but not with microvilli in polarized tracheal epithelial cells.

### PAM associates directly with filamentous actin

**Figure 7:**
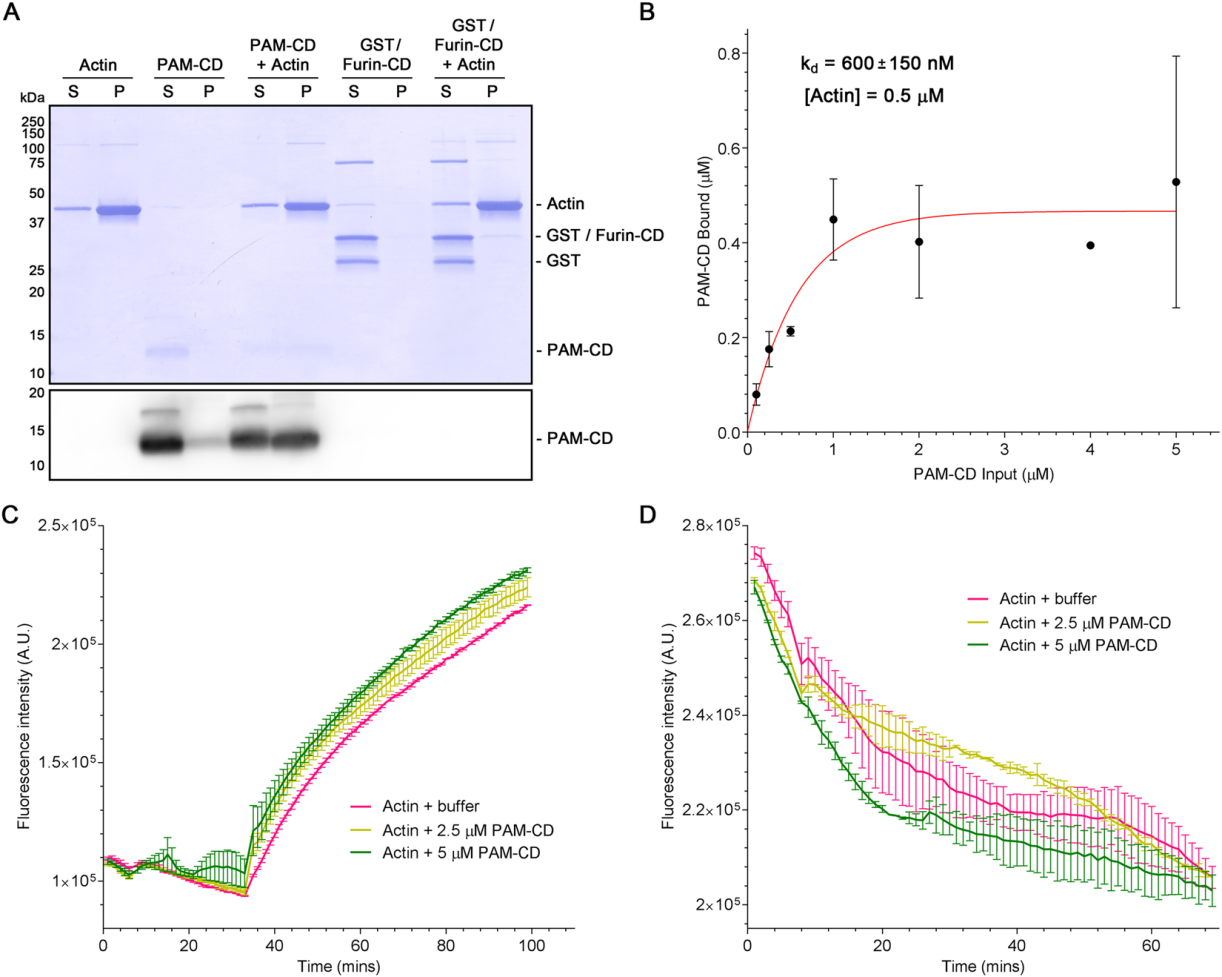
PAM interacts directly with actin but does not alter filament assembly/disassembly kinetics. (**A**) Filamentous actin (5 μM) and PAM-CD (10 μM) were incubated together or with buffer alone, and then sedimented. Supernatants and pellets were electrophoresed and stained with Coomassie blue; as the PAM-CD stains poorly with Coomassie blue, an immunoblot for this protein is also shown. In the absence of actin, nearly all the PAM-CD remained in the supernatant. However, in the presence of F-actin, approximately 50% of the PAM-CD was found in the pellet. In contrast, a control protein, GST/Furin-CD (also used at 10 μM), did not co-sediment with F-actin. The band at ~75 kDa in the GST/Furin-CD lanes is a contaminant that copurified with the fusion protein; the very minor ~110 kDa band present in the filamentous actin samples is α-actinin (AKL99 datasheet; Cytoskeleton Inc.). (**B**) Increasing concentrations of PAM-CD were incubated with 0.5 μM filamentous actin and the amount of PAM-CD present in the supernatants and pellets determined. The binding curve was fitted using a nonlinear regression single-phase exponential. The dissociation constant (k_d_) was 600 ± 150 nM (best-fit Scatchard analysis; n = 3). (**C**) The effect of PAM-CD (2.5 and 5 μM) on the rate of polymerization was determined using a fluorescent pyrene-actin assay with 5 μM G-actin. After an initial 30-minute incubation, polymerization was initiated by the addition of ATP; differences were not significant in a one-way ANOVA; P = 0.364. (**D**) PAM-CD (2.5 and 5 μM) was bound to pre-formed pyrene-actin filaments (5 μM), and the sample then placed under depolymerizing conditions; differences were not significant in a one-way ANOVA; P = 0.678.

As PAM colocalizes with apical actin in polarized ciliated airway cells and can affect the actin cytoskeleton through interactions with Rho-GEFs^39^, we examined whether PAM could also associate directly with filamentous actin. Purified recombinant rat PAM-CD was incubated in the presence or absence of preassembled rabbit muscle ATP-actin filaments followed by high speed centrifugation. In the absence of actin filaments, most of the PAM-CD remained in the supernatant. However, in the presence of actin filaments, PAM-CD was found with F-actin in the pellet; control proteins, including a glutathione S-transferase (GST)/Furin-CD fusion protein, remained in the supernatant (Fig. 7A). To determine the affinity of this interaction, we used the standard F-actin spin-down approach^40^. Varying concentrations (0.1–5 μM) of purified PAM-CD were incubated with a constant amount of F-actin (0.5 μM) and the amount of PAM-CD that co-sedimented with filamentous actin was determined by electrophoretic analysis; based on three experiments, the affinity constant (k_d_) for this interaction was 600 ± 150 nM (Fig. 7B).

We next examined the effect of PAM-CD on the polymerization of G-actin and the depolymerization of F-actin using fluorescent pyrene-actin assays^41^. The presence of PAM-CD had little effect on the rate of G-actin polymerization (Fig. 7C) and did not alter the depolymerization kinetics of F-actin (Fig. 7D). Thus, PAM is a high-affinity F-actin binding protein that could potentially tether PAM-containing vesicular structures to the actin cytoskeleton but does not directly affect actin filament dynamics.

### PAM deficiency alters the actin cytoskeleton in *Chlamydomonas*

**Figure 8:**
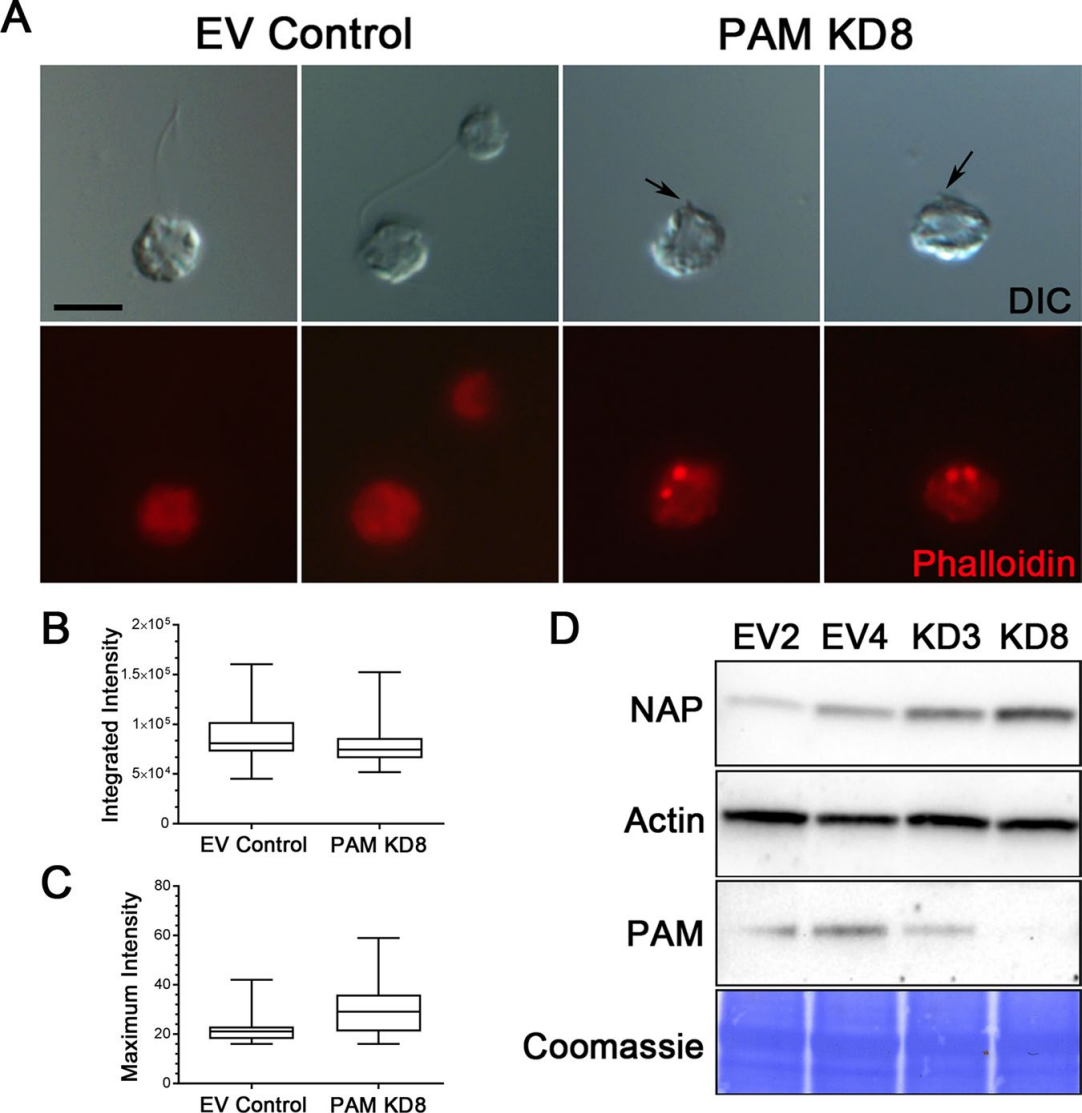
PAM-deficient *Chlamydomonas* have altered actin organization and upregulate levels of an actin paralogue. (**A**) Differential interference contrast (DIC) and fluorescence micrographs of empty vector (EV) control and PAM-deficient (PAM KD8) *Chlamydomonas* cells stained with Bodipy-phalloidin. The ciliated control cells exhibited diffuse cytoplasmic filamentous actin staining (red); no signal was present in the cilia. The PAM amiRNA cells, which only formed small ciliary stubs (arrows), show several bright foci or patches of filamentous actin staining in the cell body. (**B**) Integrated fluorescence intensities of Bodipy-phalloidin stained EV control and PAM KD8 cells were not significantly different in a two-way ANOVA (n = 19; P = 0.178). (**C**) Maximum fluorescence intensity of PAM KD8 cells was significantly different from EV controls in a two-way ANOVA (n = 19; P = 0.009). For the plots in both (**B**) and (**C**), the horizontal bar is the mean, boxes represent 95% confidence intervals, and whiskers show minimum to maximum range. (**D**) Immunoblots of cell lysates from two EV and two PAM-KD strains; equal protein loading was shown by Coomassie blue stain. The PAM-KD strains had reduced levels of PAM, whereas the canonical actin content was not altered. In contrast, levels of the actin paralogue, NAP, were significantly increased in the PAM-KD strains.

We previously demonstrated that knockdown of PAM in the unicellular green alga *Chlamydomonas* reduced PAM enzymatic activity to ~10% of empty vector control levels; these PAM-deficient cells are unable to build cilia and only assemble short ciliary stubs lacking axonemal structures beyond the transition zone^14^. Furthermore, the peri-basal body localization of intraflagellar transport (IFT) proteins required to assemble cilia is disrupted in these knockdown cells. As our biochemical data support a direct high-affinity interaction of PAM with F-actin, and as overexpression of PAM remodels the actin cytoskeleton in mammalian cells^12^, we set out to determine if alterations in the actin cytoskeleton accompanied the aberrant localization of IFT components in PAM-deficient *Chlamydomonas*. We stained control and PAM amiRNA *Chlamydomonas* cells with fluorescent phalloidin. Control cells displayed a mostly diffuse cytoplasmic staining, sometimes with a stronger perinuclear signal; the cilia, which contain inner arm dynein-associated actin monomers^42^ and presumably actin involved in ciliary ectosome release^29,30^, were not detectably stained (Fig. 8A). However, in PAM knockdown cells, bright foci of phalloidin-bound filamentous actin were visible in the cytoplasm, often located close to the ciliary stubs (Fig. 8A). The total integrated Bodipy-phalloidin fluorescence intensity in control and PAM knockdown cells was not significantly different (P = 0.18; Fig. 8B), suggesting that the total amount of filamentous actin in the cytoplasm of these strains was essentially unchanged. However, consistent with the observed fluorescent patches, there was a significant change in the maximum fluorescence intensity (P = 0.009; Fig. 8C) between control and PAM knockdown cells, further indicating that the actin cytoskeleton had been reorganized.

A *Chlamydomonas* mutant (*ida5*) lacking actin is viable^43^, as it upregulates expression of NAP, an actin paralogue which partially compensates for the lack of canonical actin^42–44^. To test whether PAM deficiency altered expression of actin and NAP, we probed cell lysates for the presence of both proteins (Fig. 8D). Although actin levels were little changed in the PAM knockdown strains compared to controls, there was a significant increase in NAP levels (Fig. 8D). The increase in NAP was 2.61 ± 0.58-fold (n = 5) compared to the average of empty vector controls for the KD8 strain which has ~10% of normal PAM activity, and 2.38 ± 0.37-fold (n = 5) for the KD3 strain which has ~30% of normal PAM activity (and see ref.^14^). This suggests that *Chlamydomonas* respond to the actin reorganization that results from PAM deficiency by upregulating NAP expression, which also occurs with, and generally compensates for, the complete loss of conventional actin in *ida5* mutant cells.

Together, these data from three model systems indicate that a functional interaction between PAM and the actin cytoskeleton has been conserved between metazoans and the evolutionarily distant chlorophyte algae.

## Discussion

In this report, we utilized evolutionarily distant model organisms to test the significance of a link between PAM and cytoskeletal-based cellular extensions (cilia and microvilli). The highly conserved features shared by PAM in *Chlamydomonas*, zebrafish and mice include membrane topology, consumption of molecular oxygen and ascorbate, and dependence on copper. Zebrafish *pam*^−/−^ embryos exhibit cardiac and gut edema, small eyes, cystic kidneys, hydrocephalus, the loss of both actin-based microvilli and ciliary structures in the pronephros, and ultimately die with massive edema. Our analysis of murine tracheal and *Chlamydomonas* cells revealed a functional association between PAM and the actin cytoskeleton that has been conserved across a broad phylogenetic range. Our biochemical assays demonstrated a direct, sub-μM affinity interaction between rat PAM-CD and filamentous actin.

Zebrafish express Pam in numerous ciliated tissues during early development including the ependyma lining the brain ventricles, otic vesicles and pronephros; notably, Pam is not expressed in the zebrafish heart at these early time points. Nevertheless, *pam*^−/−^ zebrafish embryos recapitulate the edematous mutant mouse phenotype, ultimately leading to lethality after ~10 days. Although *pam*^−/−^ zebrafish appear morphologically normal up until 48–72 hpf, incipient edema may affect the stability of cellular extensions such as microvilli and cilia. Deletion of the mouse *Pam* gene causes gross edema and mid-embryonic lethality^8^, although the source of this edema, first observed in the cardiac region early in development, is unknown. In addition to the vasculature alterations and ventricular hypertrophy observed in the PAM-null mice, the edema may result from altered fluid homeostasis caused by hormonal dysregulation^8^.

Zebrafish *pam*^−/−^ embryos display several striking phenotypes including hydrocephalus and cyst-like structures, which point to ciliary dysfunction in the brain and pronephros, respectively. For example, similar defects were observed in zebrafish morphants that disrupt the assembly of ciliary outer dynein arms or the nexin-dynein regulatory complex, and thus have compromised ciliary motility and fluid flow^18^. Unlike mutants/morphants that directly impact ciliary motility *per se*^17,18,45,46^, the *pam*^−/−^ zebrafish embryos do not exhibit laterality defects, consistent with the presence of cilia at early developmental stages. Similarly, motile cilia are involved in normal otolith biomineralization, *e.g*.^24,47^, although these motile organelles are not absolutely essential for otolith formation or tethering^48^. We observed no otolith abnormalities in the *pam*^−/−^ embryos, and both actin-based stereocilia and kinocilia, which assemble on the sensory hair cells early in development at the 19 somite stage (~16 hpf), were present^48^.

Several studies have pointed towards a connection between PAM and the actin cytoskeleton. The ability of the PAM-CD to interact with Kalirin and Trio, multidomain proteins known to regulate actin cytoskeletal organization through their Rho-GEF and phosphatidylinositol-binding Sec. 14 domains, is thought to play an essential role in the ability of PAM to affect neuroendocrine cell morphology^11,39^. Overexpression of PAM in murine neuroendocrine cells alters the actin cytoskeleton and inhibits the regulated exocytosis of secretory vesicle content^12^. Here we find that the absence of Pam leads to the loss of actin-based microvilli in zebrafish embryos, and that reducing PAM expression dramatically alters actin cytoskeletal organization in *Chlamydomonas*, thereby revealing an evolutionarily conserved functional association between PAM and actin. Intriguingly, the PAM-CD is not highly conserved (Supplemental Fig. S7), suggesting that species-specific interactions may accomplish similar tasks in different organisms.

Using biochemical assays, we found that recombinant PAM-CD exhibits specific binding to filamentous actin with a dissociation constant in the sub-μM range, suggesting that the association is physiologically relevant; indeed, the measured k_d_ (600 ± 150 nM) is essentially identical to that obtained previously for the binding of chicken smooth muscle α-actinin to actin^49^. Binding of PAM-CD did not alter the polymerization of G-actin or the disassembly kinetics of actin filaments *in vitro*; however, this interaction might potentially tether PAM-containing membranous structures to the actin cytoskeleton, and thereby allow them to be concentrated in the apical region of polarized cells. This possibility is reinforced by our analysis of multiciliated tracheal epithelial cells where PAM and phalloidin-staining co-localize near the apical surface in the peri-basal body region, which also contains numerous membrane-bound vesicles many of which are likely destined for the ciliary and/or microvillar membranes. Notably, although PAM is present in the membrane of motile cilia on tracheal cells^13^, it does not localize to the microvillar membrane.

Previously, we observed that the Golgi stacks in PAM-deficient *Chlamydomonas* cells are more curved than in control cells^14^. Golgi architecture is compromised by actin-depolymerizing drugs such as the latrunculins^50–52^, and disruption of actin-binding also leads to abnormal Golgi morphology^53–56^. Thus, the changes in the Golgi architecture of PAM-deficient *Chlamydomonas* may be a consequence of the actin cytoskeletal reorganization that we have now found occurs in these strains.

The actin cytoskeleton has been implicated in the assembly of both primary and motile cilia^28,38^. In primary ciliated cells, actin has been suggested to regulate ciliogenesis through the transcriptional co-activators YAP/TAZ and vesicular trafficking^27^. Furthermore, the microRNA miR-129 promotes primary ciliogenesis by inhibiting both expression of the centriolar-capping protein CP110 and the formation of branched F-actin structures^57^. Similarly, low levels of cytochalasin D that are insufficient to disrupt stress fibers also promote ciliogenesis, potentially by affecting a highly dynamic subset of F-actin^28^. This change in actin cytoskeletal organization is thought to act as a switch promoting ciliogenic trafficking^58^. Furthermore, actin controls the release of ciliary ectosomes during G protein-coupled receptor signaling^29^, and actin polymerization leads to excision of the ciliary tip and loss of IFT components prior to ciliary resorption^30^. In *Chlamydomonas*, inhibition of actin polymerization with latrunculin B results in shortening of preformed cilia by affecting the entry of IFT components into the organelle^59^. Furthermore, in the absence of the actin paralogue NAP, latrunculin affects protein synthesis, ciliary protein assembly and transition zone component localization^60^. Thus, although there is a clear functional connection between actin and ciliary assembly and maintenance, the interplay between these systems is complex and varied.

In Pam-null zebrafish, we find a striking loss of microvilli in the pronephros which precedes the loss of cilia, suggesting that alterations in the actin cytoskeleton might contribute to defective ciliogenesis. Furthermore, we observed the formation of cytosolic axonemes lacking a ciliary membrane and the presence of basal bodies within the cytoplasm rather than docked at the plasma membrane. A similar cytosolic axoneme assembly phenotype was seen previously in PAM-deficient planaria^14^. We propose that the lack of PAM leads to changes in actin cytoskeletal organization, and consequently disrupts basal body attachment and ciliary membrane formation while leaving axonemal assembly unaffected (Supplemental Fig. S8). Loss of PAM might also lead to enhanced branched actin polymerization at the ciliary tip causing increased ciliary ectocytosis and consequent ciliary membrane loss without altering axonemal architecture^27,29^.

We previously demonstrated that the amidating activity of PAM is a key requirement for ciliogenesis^14^. Indeed, treatment of deciliated wildtype *Chlamydomonas* with either a specific mechanism-based PHM inhibitor (4-phenyl-3-butenoic acid) or neocuproine, a copper-specific metal chelator (PHM absolutely requires copper for activity), significantly delayed the reformation of full-length cilia^14^. In Pam-null zebrafish, we observed that ciliary loss did not occur until mid-developmental stages even though there is no Pam enzymatic activity in these embryos by 48 hpf. This indicates that there are no maternally-derived stores of Pam enzyme in the *pam*^−/−^ homozygotes at 48 hpf, and thus no amidated peptides can be generated by these embryos at or beyond this point in development. So why can Pam-null embryos form cilia at early time points, but not later in development? Although this remains to be determined, there is maternally-contributed *pam* mRNA in the zygote at ~4 hpf (see supplementary data in ref.^61^). Thus, some Pam enzyme may be present at this very early developmental stage and could potentially generate sufficient amidated products to allow ciliogenesis to occur for several days. Therefore, cilia that form early in development may assemble normally, while tissues that become ciliated at later times, or in which cilia undergo continual extensive remodeling or exhibit increased ectocytosis, are defective. Furthermore, in other fish species (*e.g*. the tropical damselfish, *Pomacentrus amboinensis*), maternally-derived hormones present in the yolk have been shown to impact the rate of embryonic development^62^. Thus, zebrafish embryos may have a stock of maternally-derived amidated products stored in the yolk sac, which provides nutrition until ~ 5 dpf; failure of ciliogenesis would begin only after this store of peptides was depleted. In both these scenarios, Pam-generated amidated products, and thus cilia, would be present during early development, allowing for the normal determination of laterality and otolith biomineralization.

Collectively, our data from Pam-null zebrafish, ciliated murine tracheal cells and PAM-deficient *Chlamydomonas*, combined with *in vitro* biochemical assays, suggest a model whereby PAM coordinates actin and ciliary assembly during development.

## Materials and Methods

### General zebrafish care and maintenance

All work involving zebrafish (*Danio rerio*) embryos and adult fish was performed at the Marine Biological Laboratory and approved by the Institutional Animal Care and Use Committee under protocol number 16–36. All embryos were collected from single-pair matings and raised at 28.5 °C in egg water (60 µg/ml Instant Ocean stock salts (Pentair) in system water)^63^. Zebrafish strains used in this study were AB (wild type). All embryos were staged by either hours post-fertilization (hpf) or days post fertilization (dpf), and by morphological criteria based on the zebrafish staging chart^64^.

### *In situ* hybridization

*In situ* hybridization experiments with digoxigenin-labeled sense and antisense *pam* probes were performed as described previously^65^. The *pam* probes were synthesized from 72 hpf embryo cDNA using custom-designed primers: forward 5′-CCATGCCAGTATGGACACAG-3′ and reverse 5′-TGTGTTGGTGGCTGGATAAA-3′.

### CRISPR/Cas9 gene editing

#### Single guide RNA (sgRNA) generation

The sgRNA guide sequence TAGTCACAGTATCCAAAACC (Integrated DNA Technologies) was designed adjacent to the protospacer adjacent motif sequence (CCA). The sgRNA template was prepared as described^66,67^, and PCR products were purified using Qiagen PCR purification columns (Qiagen 28104). *In vitro* transcription of the sgRNA was performed using a Megascript T7 transcription kit (Ambion AM1334), and sgRNA was purified using mini Quick Spin RNA Columns (Roche 11814427001).

#### Injections

To induce targeted mutagenesis in exon 1 of *pam*, 40 ng/µl sgRNA was combined with 80 ng/µl Cas9 mRNA (PNA Bio) and injected into one-cell staged AB embryos in embryo medium (Hank’s Stock #1, Hank’s Stock #2, Hank’s Stock #4, Hank’s Stock #5, Hank’s Stock #6, double-distilled H_2_O, adjusted to pH 7.2 with NaOH). Embryos were reared for 24–72 hpf, and pools of embryos were collected for digestion and extraction of genomic DNA to confirm mutations had occurred; all genotyping analyses were carried out using standard protocols (see Mutation analysis below). Upon identification of mutation(s), F0 embryos were grown to adulthood (sexual maturity) and crossed with wild type (AB) fish. By the F1 generation, numerous mutants with alterations within the target sequence in Exon 1 were obtained (Fig. 2B and Supplemental Fig. 2A).

### Mutation analysis

When identifying mutants, individual embryos or adult fins were clipped and the tissue digested for 3 hours at 55 °C in 0.150 mL lysis buffer (10 mM Tris.Cl pH 8.0, 10 mM NaCl, 10 mM EDTA, and 2% SDS) with 20 mg/ml Proteinase K (Sigma). DNA was isolated by ethanol precipitation and PCR conducted with Phusion polymerase (New England Biolabs) using the manufacturer’s protocol. Initial identification of mutations was determined utilizing the T7 endonuclease assay (New England Biolabs). Primers used for PCR were:

forward 5′-ATTGCTTATGGAGGAGGAGG-3′ and

reverse 5′-TAAGATGGACTTCTGAATTTAAATGTTTG-3′.

Following the reaction, samples were run on 2% agarose gels and PCR product sizes determined. DNA sequencing was performed by GeneWiz (South Plainfield, NJ).

### Light microscopy of zebrafish embryos

All embryos were either fixed with 4% paraformaldehyde in phosphate-buffered saline or anesthetized in Tricane (4.2% working stock; Sigma). Both fixed and live embryos were examined and imaged using an Olympus SZX12 dissecting microscope and camera. For immunofluorescence analysis, embryos were labeled with primary antibody against acetylated tubulin (clone 6-11-B1; Sigma-Aldrich) followed by Alexa Fluor 488-conjugated goat anti-mouse antibody (A32723; Invitrogen), and imaged using a Zeiss LSM 710 confocal microscope.

### Enzyme assays of zebrafish lysates

To prepare zebrafish embryo lysates for enzyme assays, deyolked embryos were homogenized in low ionic strength buffer (20 mM Na TES pH 7.4, 10 mM mannitol) containing 1% Triton X-100 (Surfact-Amps^TM^; Thermo Scientific) and protease inhibitors^68^. Following two rounds of freeze-thaw, samples were incubated at 4 °C for 20 min and centrifuged at 18,000 × g for 15 min at 4 °C to collect solubilized proteins. PHM and PAL enzyme assays were performed at pH 5.5 using 1.0 μg protein and 5 μM CuSO_4_, as described^68^.

### Electron microscopy of zebrafish embryos

Electron microscopy of zebrafish embryos was performed essentially as described previously^69^. Following glutaraldehyde fixation, samples were post-fixed in 1% osmium tetroxide, 0.8% potassium ferricyanide in 0.1 M sodium cacodylate buffer prior to dehydration and embedding in Poly/Bed 812. Embryos were oriented so that transverse ultra-thin sections (70 nm thick) through the pronephros were obtained. Sections were stained with 6% methanolic uranyl acetate and visualized in a Hitachi H-7650 transmission electron microscope operating at 80 kV. To follow the entire pronephros, thick sections (~0.5 μm thick) of resin-embedded embryos were stained with 1% toluidine blue and imaged by bright-field microscopy; ultra-thin section were then obtained at intervals of ~50 μm.

### *Chlamydomonas* cell culture

*Chlamydomonas* cells transformed with plasmids containing amiRNA sequences directed against CrPAM and the empty vector controls were described previously^14^. Cells were grown in TAP medium^70^ under constant illumination with continual shaking.

### Immunoblot analysis of *Chlamydomonas* lysates

*Chlamydomonas* cell lysates were prepared by resuspending cells in 20 mM NaTES pH 7.4, 10 mM mannitol, 1% Triton X-100 (TMT) buffer containing 0.2 M NaCl and protease inhibitors, followed by two-rounds of freeze-thaw and sonication. Samples were centrifuged at 9,500 × g for 2 min at 4 °C, and the soluble fraction immunoblotted as described previously^14^; this procedure solubilizes essentially all PAM protein. Antibodies used were mouse monoclonal actin antibody (GTX14126; GeneTex) 1:1,000, and rabbit polyclonal antibodies against NAP^44^ 1:5,000, and CrPAM-CD^13^ 1:1,000.

### Immunofluorescence microscopy

All procedures involving mice were approved by the University of Connecticut Health Center Institutional Animal Care and Use Committee (protocol 101529-1119), in accordance with National Institutes of Health and ARRIVE guidelines (https://www.nc3rs.org.uk/arrive-guidelines). Tracheal epithelial cells isolated from adult C57BL/6 male and female mice (at the level examined, no differences were observed between sexes) and *Chamydomonas* were immunostained as described previously^13,71^; for phalloidin staining, cells were fixed in 2% paraformaldehyde to avoid any methanol in the fixative. The following antibodies/stains were used: affinity-purified rabbit polyclonal PAM JH629 antibody^72^ (1:3,000), Bodipy-phalloidin (1:1,000 of 0.5 mg/ml stock in methanol; ThermoFisher Scientific), FITC-phalloidin (1:1,000 of 0.5 mg/ml stock in ethanol; Sigma-Aldrich), mouse monoclonal ezrin antibody (1:1,000) (3C12, ThermoFisher Scientific). Hoechst 33342 (ThermoFisher Scientific) was used to stain the nucleus. Differential interference contrast and fluorescent images of *Chlamydomonas* cells were obtained using an Olympus BX-51 microscope equipped with a UPlanApo 100 × /1.35 n.a. oil immersion objective and a ProgRes CFscan digital camera (Jenoptik, Jena, Germany). The integrated and maximum fluorescence intensities of Bodipy-phalloidin stained cells were measured using ImageJ. Tracheal cells were imaged using a Zeiss Axiovert 200 M with a 63× oil immersion objective and AxioVision software. Optical sections were collected with the ApoTome module.

### Conventional and immunogold electron microscopy of mouse trachea

For conventional TEM, C57BL/6 mice were anesthetized with ketamine (100 mg/kg) and xylazine (10 mg/kg) and fixed by perfusion with 2% paraformaldehyde, 2.5% glutaraldehyde in 0.1 M sodium cacodylate, pH 7.4. Trachea were then excised, rinsed with buffer, and post-fixed with 1% osmium tetroxide, 0.8% potassium ferricyanide in 0.1 M sodium cacodylate buffer. Samples were stained *en bloc* with 1% uranyl acetate, dehydrated through an ethanol series and then infiltrated with a mixture of propylene oxide and Poly/Bed 812. Following infiltration with 100% resin, blocks were polymerized at 60 °C for 48 h. Ultrathin sections (70 nm) were mounted on 200-mesh or slot Cu grids coated with formvar. Sections were stained with 6% methanolic uranyl acetate and lead citrate for 3 mins. For immunogold EM, mice were anesthetized with ketamine (100 mg/kg) and xylazine (10 mg/kg) and perfusion fixed with 4% paraformaldehyde, 0.1% glutaraldehyde in phosphate-buffered saline (PBS); tissues were rinsed three times with 0.1 M cacodylate buffer pH 7.4, dehydrated, and embedded in either LR Gold or LR white resin. Ultrathin sections (80 nm) were mounted on formvar-coated Ni grids. Sections were blocked for 15 mins with 1% BSA in PBS and then incubated overnight at 4 °C with affinity-purified anti-PAM antibody (JH629) diluted 1:100 in BSA/PBS. Following buffer rinses, grids were incubated with goat anti-rabbit IgG conjugated to 10 nm gold (#25109; Electron Microscopy Sciences) for 60 mins at room temperature. Sections were washed with buffer and then counterstained with 6% methanolic uranyl acetate for 3 mins. All grids were imaged in a Hitachi H-7650 transmission electron microscope operating at 80 kV.

### Actin binding and polymerization/depolymerization assays

Recombinant PAM-CD [rat PAM(898–976)] was purified after expression in *E. coli* BL21(DE3) using the pET-11CD vector^73^. GST/Furin-CD was bound to glutathione-Sepharose (Amersham Pharmacia Biotech) and eluted with glutathione, which was removed by dialysis^74^. Actin high-speed cosedimentation assays were performed essentially as described^75^, using pre-formed actin filaments derived from rabbit skeletal muscle (#AKF99; Cytoskeleton Inc). Centrifuges used were either a Beckman TL100 with TLA120.1 rotor at 100,000 rpm for 15 mins or a Beckman Airfuge with an A100/30 rotor operating at maximum pressure for 60 mins. For Coomassie blue staining, cosedimentation assays contained 5 μM actin final concentration and either 10 μM PAM-CD or 10 μM GST/Furin-CD fusion protein as a control. Assays to determine the dissociation constant used a constant amount (0.5 μM) of F-actin and concentrations of PAM-CD varying from 0.25–5 μM as described^40,75,76^. Pyrene-actin polymerization and depolymerization assays were performed using a pyrene-actin assay kit (#BK003, Cytoskeleton Inc) according to the manufacturer’s instructions.

### Statistical analyses

Data for the binding of PAM-CD to actin were fit to a non-linear single-phase exponential with goodness-of-fit R^2^ = 0.704. The dissociation constant of 600 ± 150 nM was calculated from Scatchard plots of three experiments with best-fit straight lines. Effects of PAM-CD on the rate of G-actin polymerization and the rate of F-actin depolymerization were analyzed using one-way ANOVAs and the Brown-Forsythe test. For the polymerization assay, DF = 2, F = 1.014, P = 0.364; for the depolymerization assay, DF = 2, F = 0.389, P = 0.678. Fluorescent intensities of phalloidin-stained *Chlamydomonas* cells were analyzed using two-way ANOVAs. For maximum intensity measurements, n = 19, DF = 1, F = 8.46, P = 0.009. For integrated intensity measurements, n = 19, DF = 1, F = 1.95, P = 0.178. All the above statistical analyses were performed using GraphPad Prism or Microsoft Excel. χ^2^ tests for significant deviations from expected Mendelian ratios were calculated manually, and P values obtained from the critical values of the χ^2^ distribution table^77^.

### Sequence alignment

Zebrafish (A0A0R4IIV2), human (P19021) and *Chlamydomonas* (A0A0S2C767) PAM-CD sequences were aligned using Clustal Omega (v.1.2.4) (http://www.ebi.ac.uk/Tools/msa/clustalo/).

### Data availability

All data generated and/or analyzed during this study are included in this article and its supplementary figures.

### Ethics statement

All procedures involving zebrafish (*Danio rerio*) embryos and adult fish were performed at the Marine Biological Laboratory and approved by the Institutional Animal Care and Use Committee under protocol number 16–36, in accordance with National Institutes of Health guidelines. All procedures involving mice were approved by the University of Connecticut Health Center Institutional Animal Care and Use Committee (protocol 101529–1119), in accordance with National Institutes of Health and ARRIVE guidelines (https://www.nc3rs.org.uk/arrive-guidelines).

## Electronic supplementary material

Supplementary Figures
