# Supplementary material for: Microvillar and ciliary defects in zebrafish lacking an actin-binding bioactive peptide amidating enzyme
Source: Sci Rep. 2018 Mar 14;8:4547. doi: 10.1038/s41598-018-22732-9 (PMC5852006; doi:10.1038/s41598-018-22732-9)
Supplement: Supplementary file 1 — Supplementary Figures [file 41598_2018_22732_MOESM1_ESM.pdf]

SUPPLEMENTARY FIGURES

Microvillar and ciliary defects in zebrafish lacking an actin-binding bioactive peptide amidating enzyme

Dhivya Kumar[1#†], Rebecca T. Thomason[2#§], Maya Yankova[1,4], Jonathan D. Gitlin[2], Richard E. Mains[3], Betty A. Eipper[1,3*] and Stephen M. King[1,4*]

Departments of [1]Molecular Biology and Biophysics and [3]Neuroscience, and [4]Electron Microscopy Facility, University of Connecticut Health Center, Farmington, CT 06030, and [2]Eugene Bell Center for Regenerative Biology and Tissue Engineering, Marine Biological Laboratory, Woods Hole, MA 02543.

# These authors contributed equally to this work.

† Present address: Department of Biochemistry and Biophysics, University of California San Francisco, San Francisco, CA 94158.

§ Present address: University of Virginia, Charlottesville, VA 22904.

* To whom correspondence should be addressed: eipper@uchc.edu; sking@uchc.edu

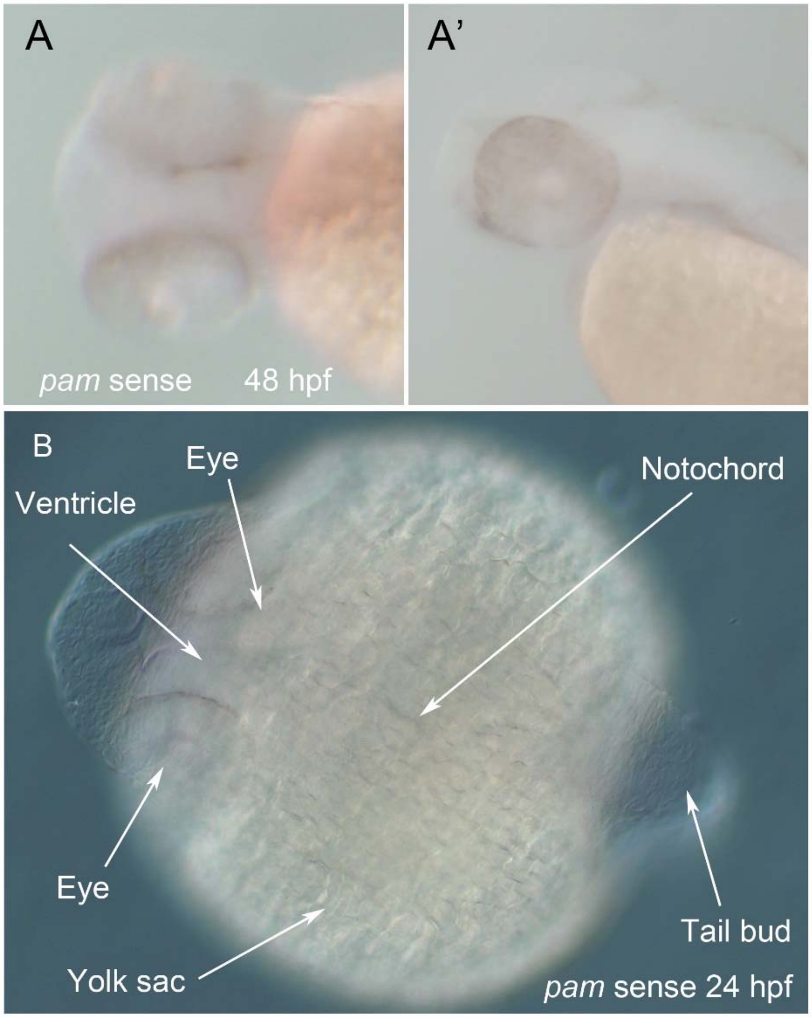

Supplemental Fig. S1 PAM *in situ* hybridization controls

Dorsal (A) and lateral (A') views of a wildtype 48 hpf zebrafish embryo subject to *in situ* hybridization using the *pam* sense probe, with the same photographic conditions as in Fig. 1. No signal was observed in the ventricles, otic vesicles or floor plate, confirming the specificity of the signals observed with the antisense probe shown in Fig. 1. (B) Dorsal view of a wildtype 24 hpf embryo subject to *in situ* hybridization with the *pam* sense probe; this image was acquired using DIC optics. No signal was observed in any tissue.

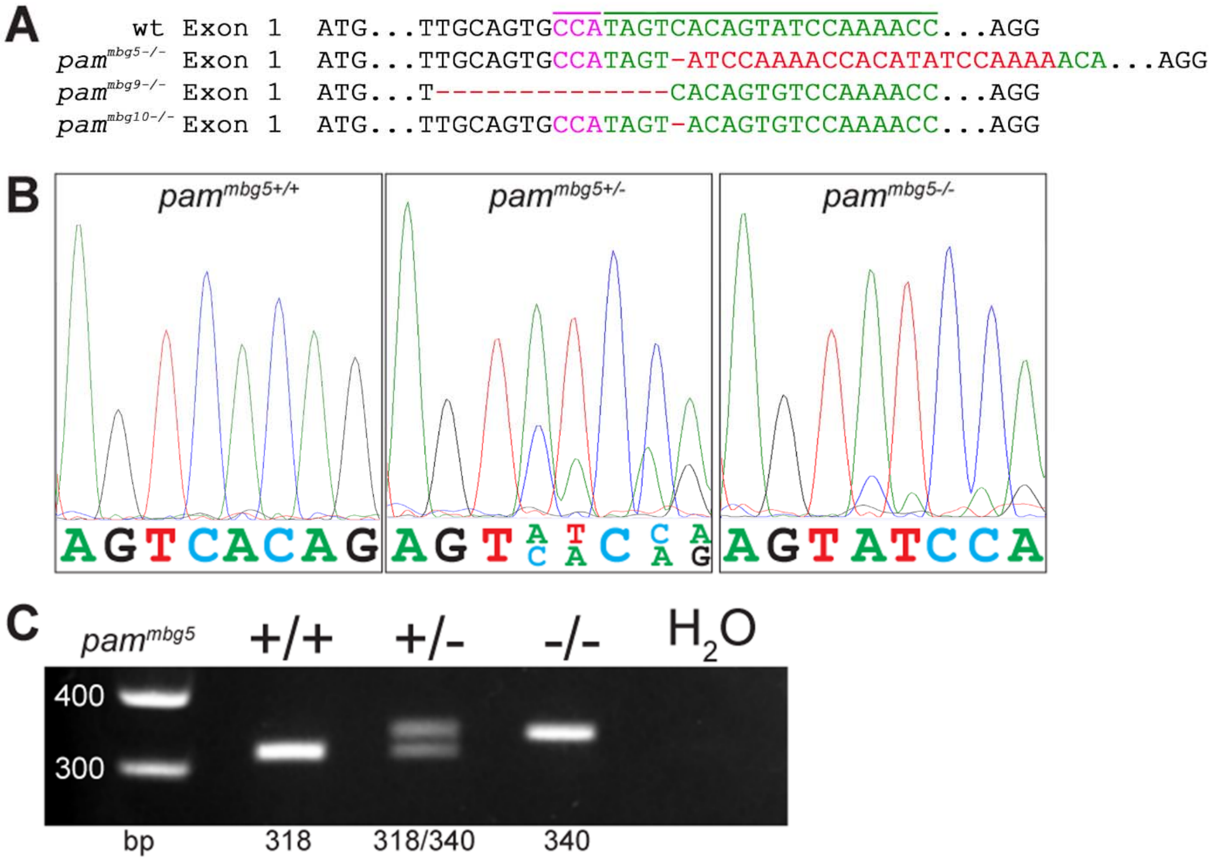

Supplemental Fig. S2 ***pam**^{mbg}* **allele sequences, and confirmation of** ***pam**$^{mbg5+/-}$* **and** ***pam**$^{mbg5-/-}$* **embryo genotypes**

(A) Sequence of the mutations in the *pam*^{mbg5}, *pam*^{mbg9} and *pam*^{mbg10} alleles generated by CRISPR/Cas9 gene editing. The protospacer adjacent motif is indicated in purple and the target sequence is in green. Sequence alterations are indicated in red; the *pam*^{mbg5} allele included a 1 bp deletion and 23 bp insertion (red). (B) DNA sequence traces of wildtype and the edited *pam*^{mbg5} hetero- and homozygotes. Both wildtype and mutant sequences are evident in the trace from the *pam*$^{mbg5+/-}$ heterozygote. (C) PCR analysis of wildtype and mutant *pam*^{mbg5} alleles yielded products of 318 and 340 bp, respectively. Both products were obtained from the *pam*$^{mbg5+/-}$ heterozygote.

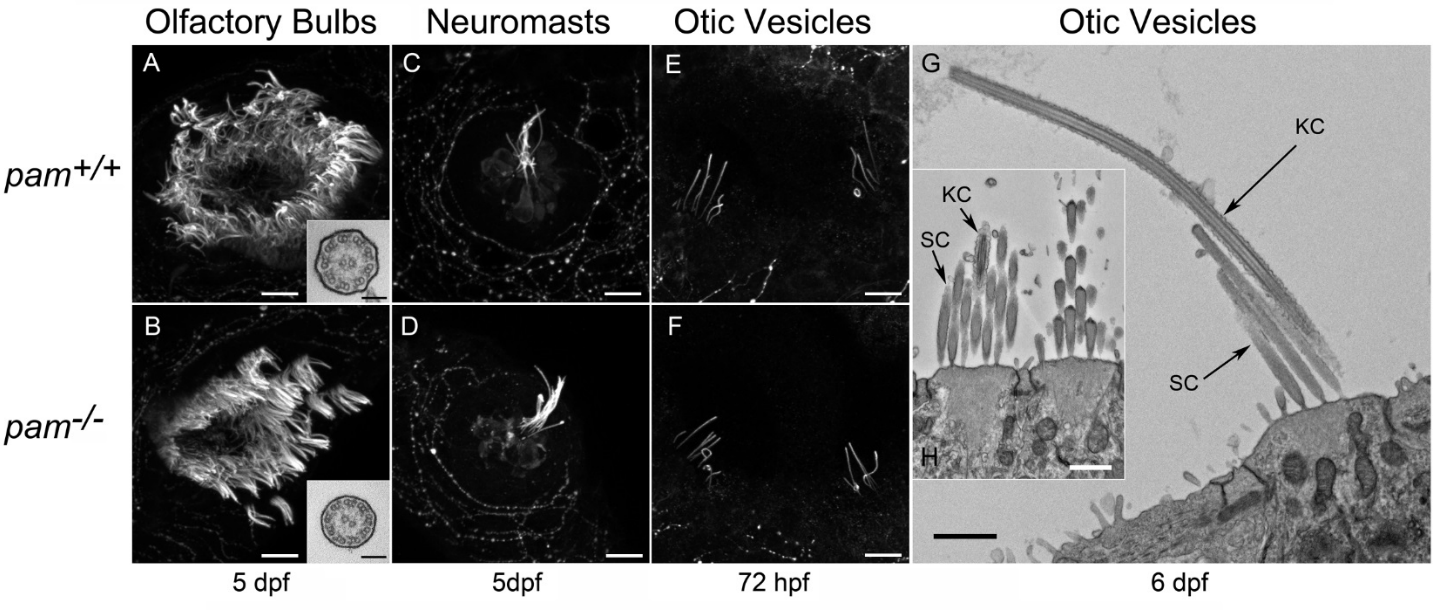

Supplemental Fig. S3 Cilia are present in the olfactory bulbs, neuromasts and otic vesicles of *pam*$^{-/-}$ embryos

Confocal immunofluorescence images of the olfactory bulb (A, B), neuromast mechano-sensory cells (C, D), and otic vesicles of control and *pam*$^{-/-}$ embryos probed with antibody against acetylated tubulin. Electron micrographs (G, H) revealed that at 6 dpf both kinocilia (KC) and actin-based stereocilia (SC) were evident emanating from the sensory hair cells in the otic vesicles of *pam*$^{-/-}$ embryos; these structures assemble early in development at ~16 hpf. The insets in (A, B) show that the olfactory motile cilia have normal 9+2 ultrastructure. Bars = 5 μm (A-F), 1 μm (G, H) and 100 nm (insets in A and B).

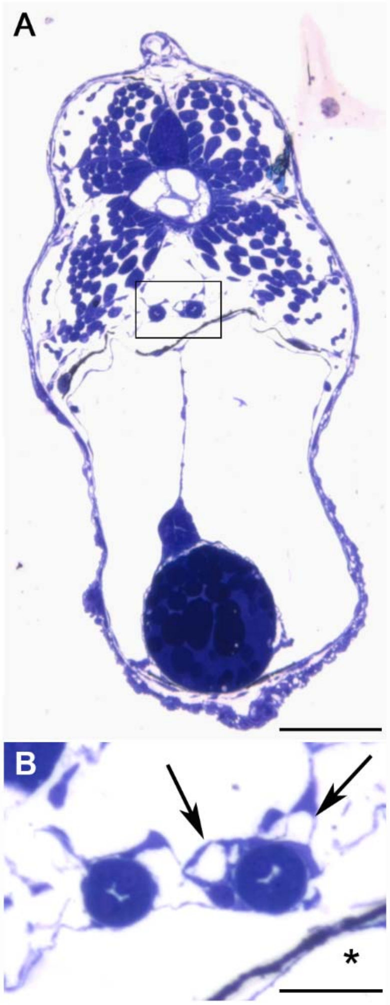

Supplemental Fig. S4 Cyst-like structures and edema in 6 dpf *pam*$^{-/-}$ embryos

Toluidine blue-stained transverse section through a 6 dpf *pam*$^{-/-}$ embryo. Massive edema was evident (*) as were cyst-like structures (arrows) which were routinely observed associated with the pronephric tubules. The boxed region in A is shown at higher magnification in B. Bars = 10 and 2.5 μm for panels A and B, respectively.

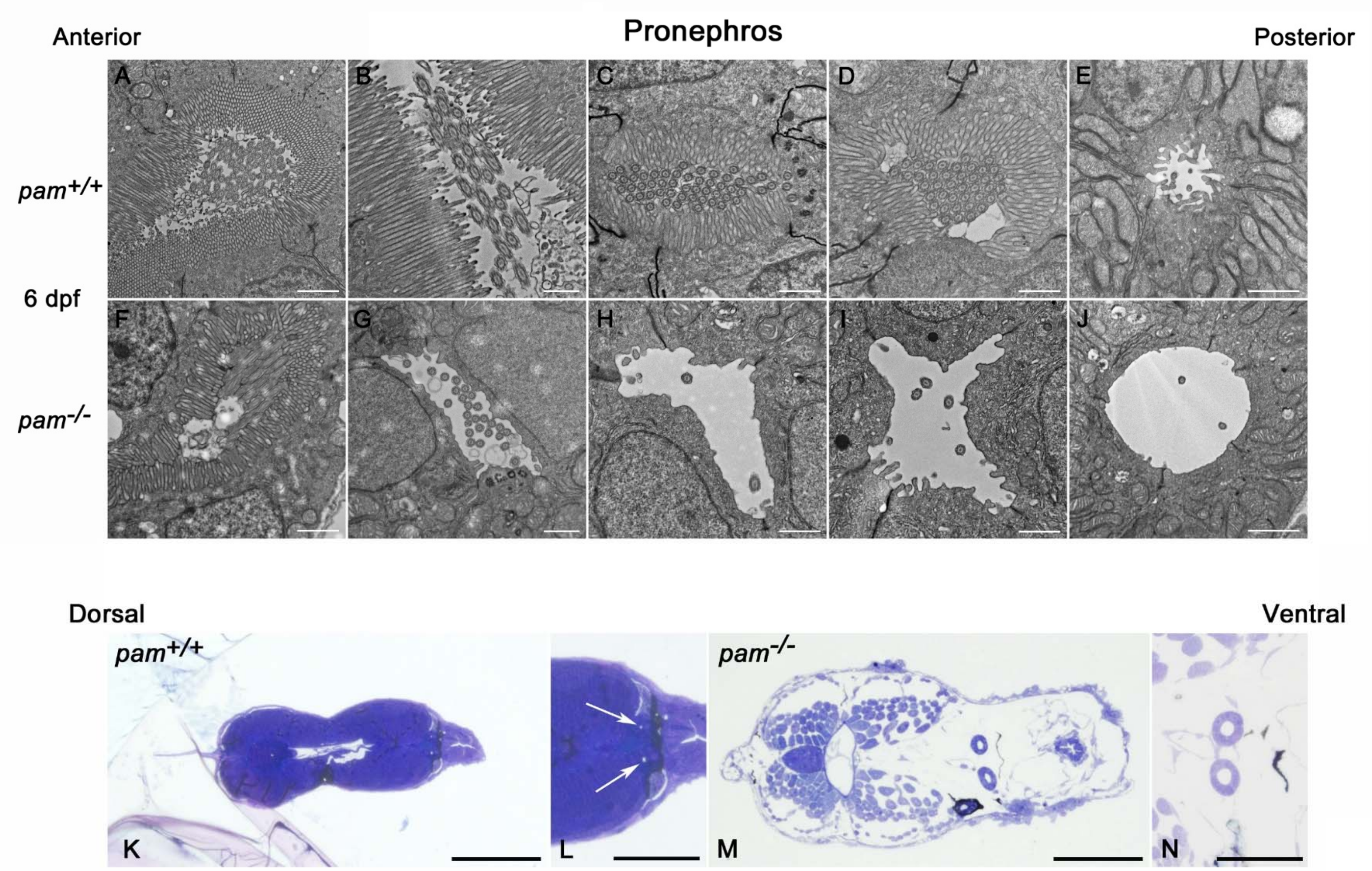

Supplemental Fig. S5 Ciliary and microvillar defects in the pronephros of 6 dpf *pam*$^{-/-}$ embryos

Upper panels - Electron micrographs of the anterior-posterior regions of the pronephros of 6 dpf *pam*$^{+/+}$ (A-E) and *pam*$^{-/-}$ (F-J) embryos. The entire pronephros was examined using alternating 0.5 μm thick sections for ~ 50 μm followed by several ultrathin sections examined by EM. Although microvilli and cilia were present in the most anterior segment of the pronephros of both wildtype and Pam-null embryos, cilia were much reduced, and microvilli were essentially absent in other pronephric regions of the mutant; only the very posterior pronephros lacked microvilli in the wildtype embryo. Bars = 2 μm (A, E, F, J) and 1 μm (B, C, D, G, H, I).

Supplemental Fig. S5 continued

Lower panels – Bright-field micrographs of toluidine blue-stained transverse thick sections through wildtype (K, L) and Pam-null (M, N) 6 dpf embryos; the vastly different appearance of the mutant embryos is due to the massive edema. Higher magnification images of the pronephric region (L, N) revealed that the pronephros lumen (white arrows in L) in the *pam*$^{+/+}$ embryo was almost occluded, whereas it was more open in the *pam*$^{-/-}$ animals. Bars = 10 (K, M) and 5 (L, N) μm.

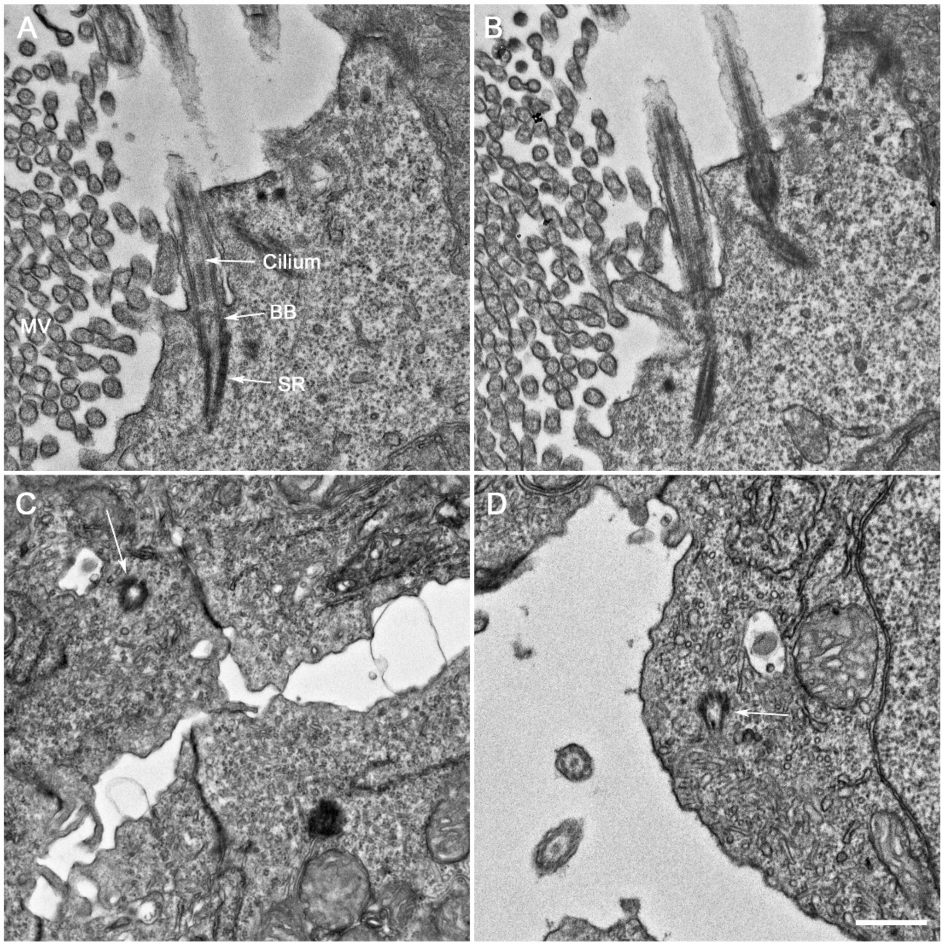

Supplemental Fig. S6 Undocked centrioles/basal bodies in 6 dpf *pam*$^{-/-}$ embryos

Transverse sections through wildtype and *pam*$^{-/-}$ embryos (6 dpf). (A, B) Adjacent sections through a cell lining the pronephros of a 6 dpf wildtype embryo showing two basal bodies (BB) with striated rootlets (SR) docked at the plasma membrane with attached membrane-bound ciliary axonemes (cilium) extending into the pronephric lumen; numerous microvilli (MV) are evident. (C, D) Undocked centrioles/basal bodies (arrows) located within the cytoplasm of cells lining the pronephros of a 6 dpf *pam*$^{-/-}$ embryo and oriented approximately parallel to the pronephros long axis as are the cytosolic axonemes (see Fig. 5H); few cilia are present in the lumen and microvilli are absent. Bar = 500 nm.

```
Cr  ARATLMHYIQSITVSSSNPGGSGAGIKGGPSAGASTPAGGGLRKTFGAWASAAEGLLGRF
Dr  ------RWKKSRMYSDD------------------------CQVK-LEPSGSAGGILGKI
Hs  ------RWKKSRAFGDS------------------------EHK----LETSSGRVLGRF
          :: :*   ...                         :        ::  :**::

Cr  SGRGGAAAAAGLAGTTGA-----GGAKRSTAEVEAARER------
Dr  RGKAAGSLNLGNFFATHKGYTRHGFDRLSTEGSDMEKDDEDATDS
Hs  RGKGSGGLNLGNFFASRKGYSRKGFDRLSTEGSDQEKEDDGSE--
     *:....   *   ::       *  : **   :  ::
```

Supplemental Fig. S7 **Sequence alignment of the PAM-CD**

The CD sequences immediately following the trans-membrane domains of *Chlamydomonas* (Cr) zebrafish (Dr) and human (Hs) PAM were aligned using Clustal Omega. Identical residues are indicated by *; high and moderate conservation are marked by ":" and ".", respectively. Colors indicate basic (pink), acidic (blue), polar (including Gly; green), and hydrophobic (red) residues.

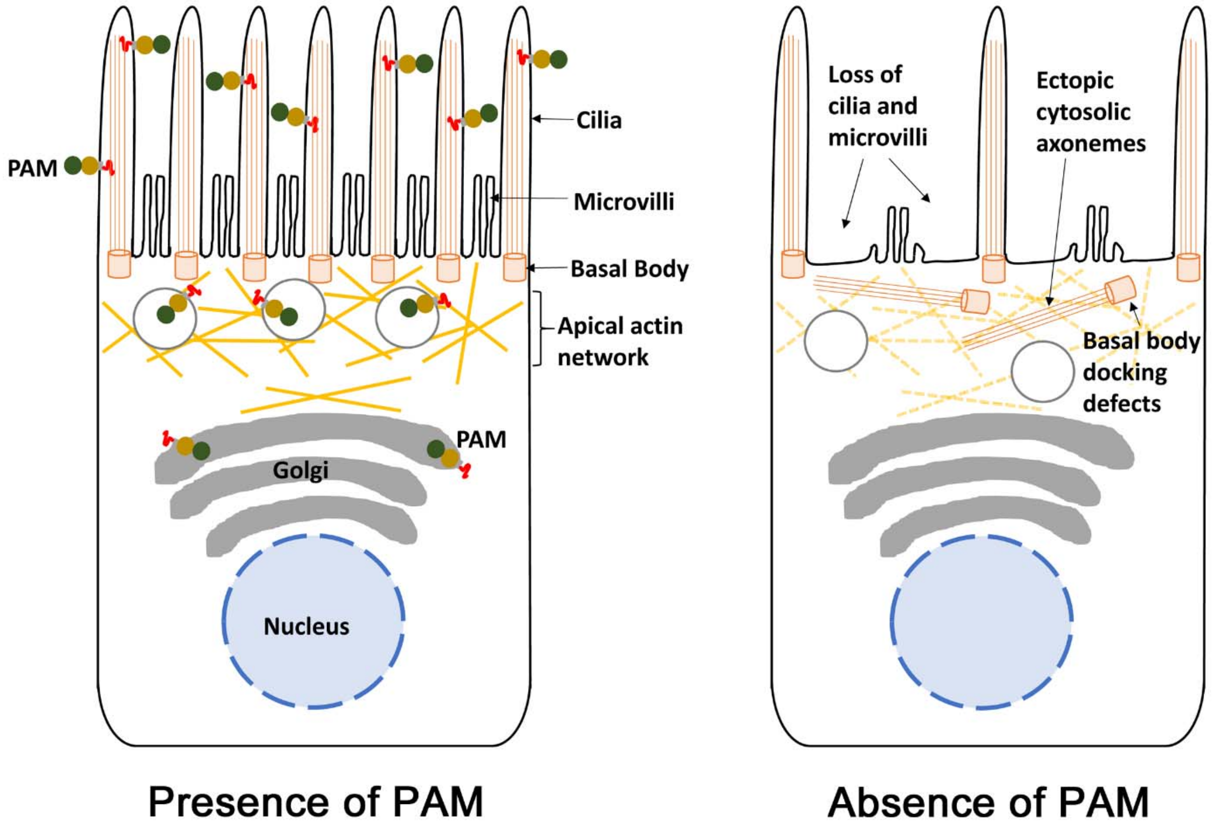

Supplemental Fig. S8 PAM controls actin cytoskeletal organization and affects basal body docking and ciliary membrane trafficking

Schematic diagram of a multiciliated cell with numerous cilia and microvilli protruding from the apical surface (*left*). PAM (green, PHM domain; orange, PAL domain; grey, transmembrane domain; red, C-terminal domain) is present in the Golgi, Golgi-derived vesicles that colocalize with the apical actin network, and cilia. The lack of PAM (*right*) leads to alterations in actin organization, the loss of both cilia and microvilli, and basal body docking defects. Undocked basal bodies template the assembly of axonemes lacking a ciliary membrane within the cytoplasm.
